# The NIH public access policy did not harm biomedical journals

**DOI:** 10.1371/journal.pbio.3000352

**Published:** 2019-10-23

**Authors:** A. Townsend Peterson, Paul E. Johnson, Narayani Barve, Ada Emmett, Marc L. Greenberg, Josh Bolick, Huijie Qiao

**Affiliations:** 1 Biodiversity Institute, University of Kansas, Lawrence, Kansas, United States of America; 2 Center for Research Methods & Data Analysis, University of Kansas, Lawrence, Kansas, United States of America; 3 Department of Political Science, University of Kansas, Lawrence, Kansas, United States of America; 4 Florida Museum of Natural History, University of Florida, Gainesville, Florida, United States of America; 5 University Libraries, University of Kansas, Lawrence, Kansas, United States of America; 6 Department of Slavic Languages & Literatures, University of Kansas, Lawrence, Kansas, United States of America; 7 School of Languages, Literatures & Cultures, University of Kansas, Lawrence, Kansas, United States of America; 8 Institute of Zoology, Chinese Academy of Sciences, Beijing, China

## Abstract

The United States National Institutes of Health (NIH) imposed a public access policy on all publications for which the research was supported by their grants; the policy was drafted in 2004 and took effect in 2008. The policy is now 11 years old, yet no analysis has been presented to assess whether in fact this largest-scale US-based public access policy affected the vitality of the scholarly publishing enterprise, as manifested in changed mortality or natality rates of biomedical journals. We show here that implementation of the NIH policy was associated with slightly elevated mortality rates and mildly depressed natality rates of biomedical journals, but that birth rates so exceeded death rates that numbers of biomedical journals continued to rise, even in the face of the implementation of such a sweeping public access policy.

## Abstract 2

This Perspective article analyzes whether the US National Institutes of Health open access policy, which from 2008 forced open access on thousands of papers, had a detectable effect on the "demography" of biomedical journals.

The US National Institutes of Health (NIH) policy was implemented as part of the NIH mission to improve “the health of Americans by conducting and funding biomedical research that will help prevent, detect, treat and reduce the burdens of disease and disability.” The policy requires that the author’s final accepted manuscript of all NIH-funded research publications be deposited in the open repository PubMed Central within 12 months of publication. Commercial publishers facing the NIH policy predictably and publicly anticipated massive revenue losses and consequent failure of many biomedical journals [1–3]; for example:

In testifying last September in support of the bill before the Subcommittee on Courts, the Internet, and Intellectual Property Committee on the Judiciary, Martin Frank, Executive Director of the American Physiological Society (APS), insisted that the issue was not access rights but revenue streams… The NIH mandate, he argued, “risks undermining the revenue stream derived principally from subscriptions, that enables publishers to add value to research articles and to enhance readers' ability to discover and use scientists’ work.”—John Willinsky [3]

At publishers’ urging, two legislative initiatives were soon proposed to reverse the policy (Fair Copyright in Research Works Act, Research Works Act), neither of which was passed by the US Congress. We note that several of the publishers that protested most vigorously are now entering rather boldly into the world of open access (OA) publishing [4].

The NIH policy is viewed as the largest forward step in the US history of the OA movement that aims to change the scholarly communications system for the global scientific community, as well as open research results to the public that funded much of the work [5]. With executive orders from President Obama [6], all US federal agencies with research and development budgets exceeding US$100 million are in the process of implementing parallel policies such that the benefits and costs could be magnified still further. The NIH policy reversal bills failed, and the policy was implemented, with economic implications that are as yet not well understood: the proposition of prohibitive loss of profit to the publishing industry has yet to be tested quantitatively.

We assessed death and birth rates of biomedical journals as a proxy for the financial health of journal publishing; numbers of journals and numbers of papers published are known to covary positively, at least in the biomedical field [7]. To that end, it is necessary to compare their birth and survival over time with birth and survival rates for journals in other fields. A deep and careful data cleaning process (see detailed methods summary in S1–S3 Text, and full compilation of R code in S4 Text) reduced an initial collection of 784,756 item records from Ulrich’s Web Global Serials Directory to a data set with 18,372 subject-classified scholarly journals published in the US that were active in 1980 or after. About one quarter of all of the journals, 4,480, had topical subject markers classified as biomedical science. Numbers of journals active by year increased steadily though the period of analysis in this study (Fig 1). Indeed, the total number of journals more than doubled between 1980 and 2015, and these increases included biomedical journals.

**Fig 1:**
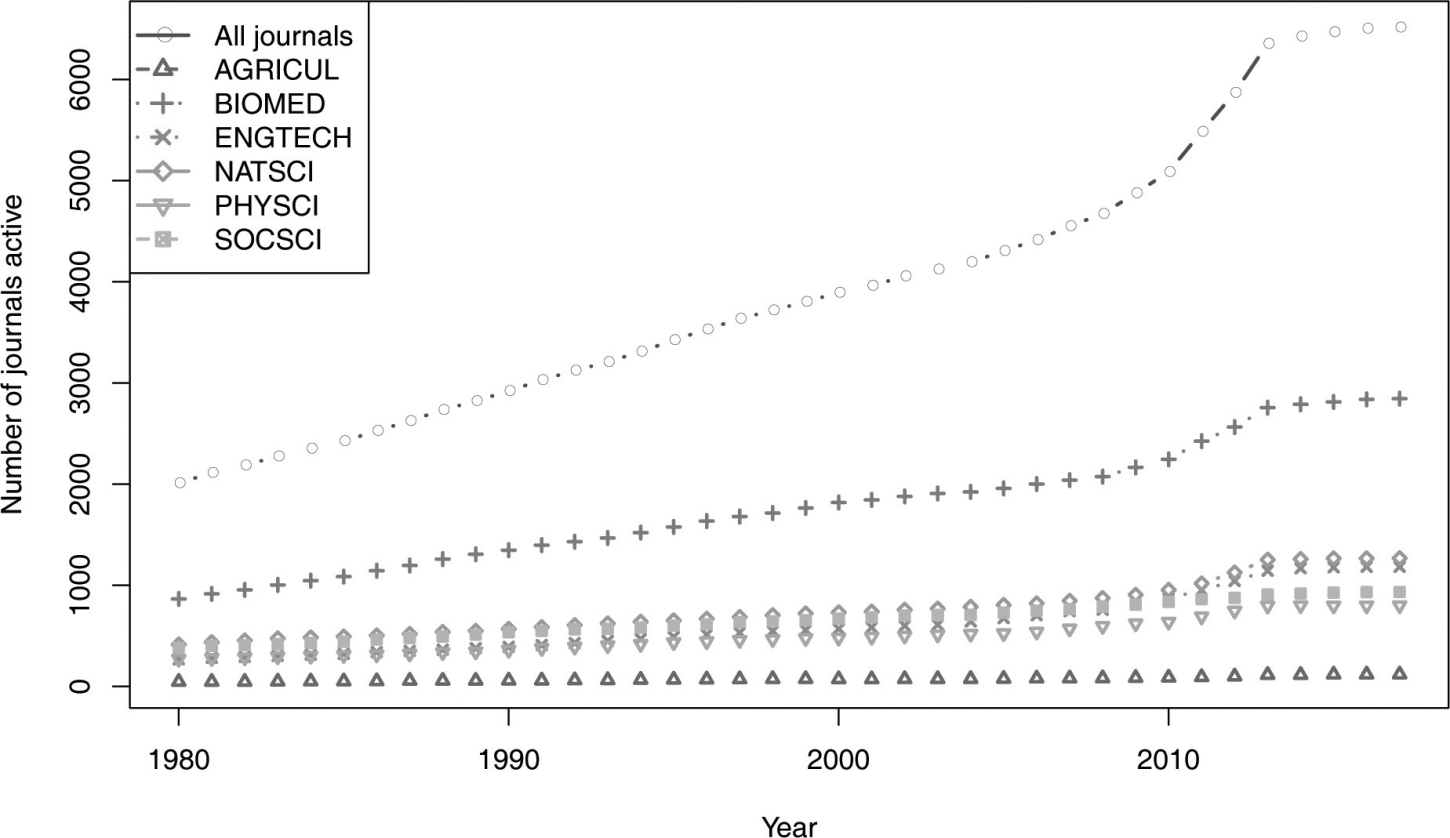
Numbers of journals active in six broad fields over the period 1990–2015. AGRICUL, Agriculture; BIOMED, Biomedical; ENGTECH, Engineering and Technology; NATSCI, Natural Sciences; PHYSCI, Physical Sciences; SOCSCI, Social Sciences.

Birth and death rates of the biomedical journals relative to rates for journals in other fields were the major focus of our analyses, which are provided in the Supporting information in the form of table summaries of yearly birth and death rates (S1 and S2 Data), respectively, for 1980–2018. However, because the data after 2015 show odd artifacts apparently unrelated to actual numbers of journals active (see below), we concentrated on 1980–2015 in our analysis (figures show trends from 1990 onward to focus on the crucial, later time period). Observed birth rates declined gradually until 2004–2005, when they jumped dramatically upward, whereas death rates were initially (1990–1998) low, moderate until 2013, and then increased dramatically thereafter (Fig 2; left-hand column).

**Fig 2:**
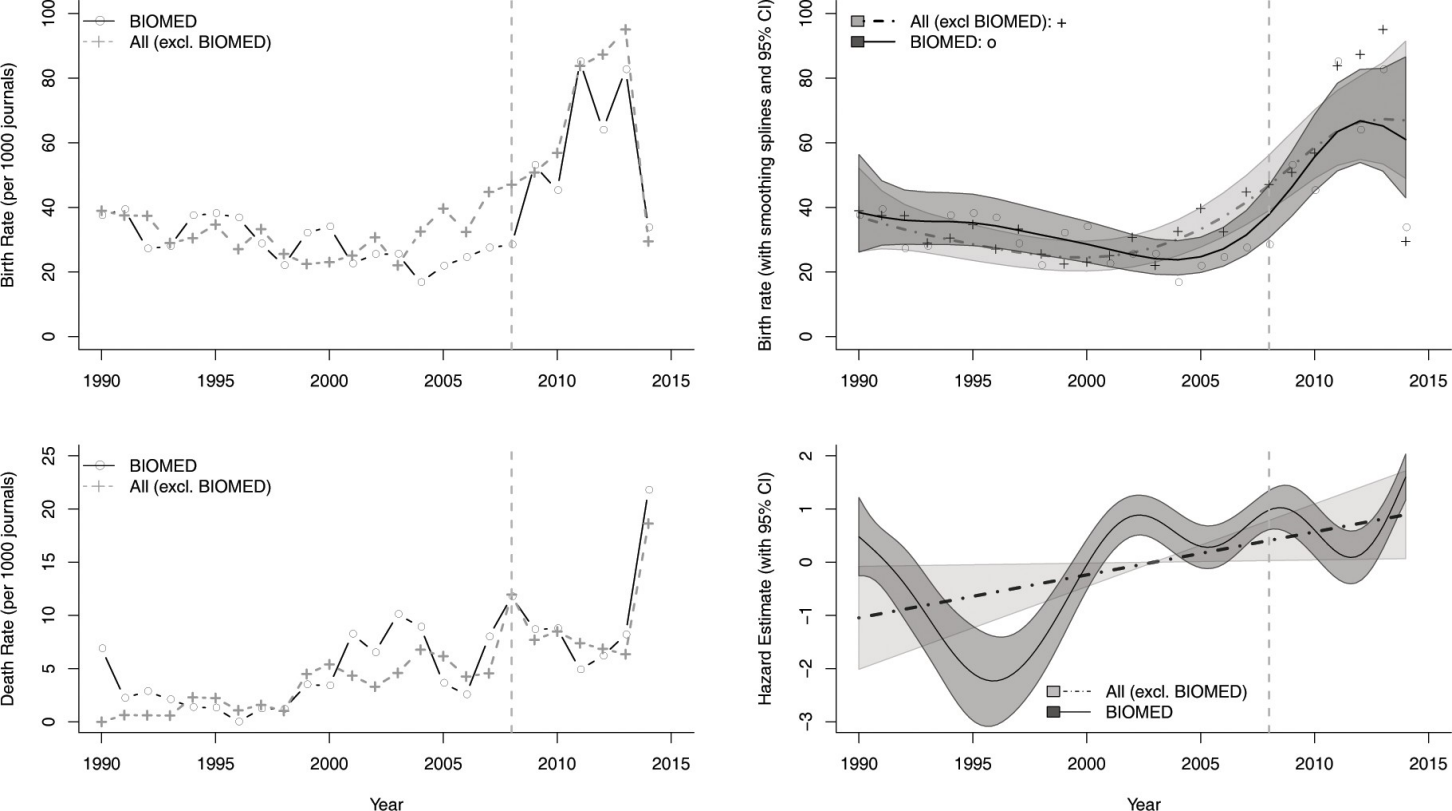
Relationship between birth and death rates in biomedical journals and in other fields. On the left-hand side, observed birth and death rates per 1,000 journals between 1990 and 2015 are presented. In the right-hand column are thin plate splines (with 95% confidence intervals in gray) that smooth year-to-year birth and death rates for observed birth rates and for hazard rate estimates from a Cox proportional hazard model, using the journal age as the timescale. The vertical reference line marks the year 2008. BIOMED, Biomedical.

A long-term, gradual decline in birth rates was apparent across all journals during 1990–2004 (Fig 2). Between 2004 and 2008, birth rates for biomedical journals were modestly depressed below those for other journals, but a dramatic rise in birth rates beginning in 2009 elevated populations of all types of journals to new heights and brought biomedical journal birth rates close to those of other subject areas. Birth rates were assessed with a generalized additive model; a thin plate smoothing spline with generalized cross-validation was estimated for each type of journal to describe effects of calendar year. The predicted values, along with approximate 95% confidence intervals associated with the smoothing splines, confirmed the contention that biomedical journal birth rates fell to a statistically significantly lower rate around 2004; however, by 2009, birth rates for the biomedical journals had recovered to match those of the other journals (Fig 2).

As regards death rates, because journal “deaths” were rare before 1990 and the data after 2015 appeared anomalous (S1 and S2 Data; see discussion below), we restricted Fig 2 to 1990–2015. Journal death rates were noticeably higher for all types of journals after 2000 (Fig 2). Estimated hazard rates from a Cox proportional hazard model using journal age as the timescale [8], treating the calendar year as a predictor, automatically accounts for age-related correlates of journal closure; we used thin plate smoothing splines to explore effects of calendar year. The hazard curve for biomedical journals showed substantial fluctuations from year to year, whereas the hazard rate for non-biomedical journals did not (the cross-validation tool indicated that a linear model was superior for the latter set). During the mid-1990s, the risk of closure for biomedical journals was lower than for other journals, but it was higher than for other journals for brief periods around 2002–2007. The confidence intervals of hazard rates of biomedical and all other journals overlapped during the time window of the public access policy change.

This paper presents a first, to our knowledge, quantitative analysis of the effects of the NIH policy on the “health” of biomedical journals, which represents a significant gap in the emerging literature on OA [9]. Using a comprehensive database of scholarly journals, we compared birth and death rates of biomedical journals over 36 years around the period of implementation of the NIH policy. We found only subtle indications of elevated death rates or depressed birth rates among biomedical journals associated with the NIH policy, likely not statistically significant, and an overwhelming increase in overall numbers of all journals analyzed over the overall study period. We analyzed the data, removing all journals indicated as OA journals in the database, as a means of removing possible biases caused by the mass appearance of the so-called predatory OA journals and obtained results that were qualitatively identical, such that our results are not an artifact of that phenomenon (see S3 and S4 Data and S1–S6 Figs). We also replicated our analyses using the PubMed Central data set, which was replete with more errors and problems and is considered inferior to and less complete than the Ulrich’s data [10,11], but obtained similar results (S5 Data, S7 Fig), such that we have some independent confirmation of these patterns from a distinct data source. As such, the journal population reduction forecasted by the publishing industry as a result of the NIH policy never occurred—to the contrary: with journal births far outnumbering deaths during the period of NIH policy implementation, the biomedical journal “population” grew massively after policy implementation and presently appears to be quite healthy; numbers of papers on biomedical topics are also known to have grown dramatically in this period [7].

A 2012 report from NIH offered similar conclusions, although apparently not based on a quantitative analysis. It cited various key points: (1) publishers enjoy a 12‐month embargo before papers are made available openly; (2) in spite of the massive downturn in the US economy over the period 2007–2011, the number of journals in biological sciences/agriculture and medicine/health increased 15% and 19%, respectively; (3) over the same period, average subscription prices of biology and health sciences journals increased by 26% and 23%, respectively; and (4) publishers forecasted increases in the medical journal market from 4.5% in 2011 to 6.3% in 2014. Hence, the report concluded that no trends in the biomedical publishing market appear consistent with broad-spectrum negative effects of the NIH policy on scholarly publishing. More broadly, the scholarly publishing industry as a whole has grown consistently in recent decades, with no indication of any marked downturn [12].

The merits of opening access to the scholarly literature are much discussed in the form of increasing citation rates and readership [13], and opening the scholarly communications universe to truly global participation [14,15]. The downsides and disadvantages of OA, however, remain little discussed and analyzed except by voices with significant conflicts of interest (e.g., publishers)—they have focused on the (nonexistent) decreased viability of the publishing enterprise [12,16] or imagined decreased quality of peer review [17].

This contribution faced a number of challenges and retains a number of limitations. Perhaps most fundamentally, we confronted a series of challenges related to data quality—the Ulrich’s data included anomalous windows of low journal birth rates in the most recent years. Indeed, in our initial analyses, which included data downloads through 2017, this birth drop-off was in 2014; in our more recent analyses, which were based on downloads a year later, the drop-off had shifted to 2015, so we are confident that this pattern represents the effects of a lag time in ingestion of “birth” data about journals in the Ulrich’s data set, a pattern that has been noted by others [18]. Replicating our analyses with an independent data set (PubMed Central) yielded results that were closely similar to those based on the Ulrich’s data (see S5 Data and S7 Fig). Most generally, this study was limited by the somewhat indirect nature of the relationship between viability of the academic publishing enterprise and the phenomena of births and deaths of journals—although a relationship certainly exists, other confounding factors enter the picture, such as the appearance of mega-journals, which cloud relationships.

To our knowledge, this contribution represents a first quantitative analysis of the proposition that OA reduces the viability of scholarly publishing endeavors. Our results indicate that the NIH policy did not accelerate the death of biomedical journals, impede new journals from appearing, or stop commercial publishers from turning massive profits [16,19], providing a quantitative basis for recent commentaries [20]. Rather, the scholarly publishing industry—including biomedical journals—is a complex, interacting system with many ongoing trends and tendencies, such as the emerging “Plan S” that could further elevate OA publishing, the emergence of so-called predatory OA journals [21], the appearance of “mega-journals” such as *PLOS ONE* and *Scientific Reports* [22], the appearance and growth of preprint archives [23], and the massive growth of East Asian science, which could serve to obscure patterns related to the NIH public access policy. In our analysis, quantitative evidence of any such negative effects were transitory at best, and the number of journals in this field has increased massively over the period that spans the implementation of the NIH policy.

## Supporting information

S1 Text Methods summary, presenting additional detail on analytical methods employed in this analysis. (DOCX)

S2 Text Summary and detail regarding data download procedures used in this study. (PDF)

S3 Text Summary and detail regarding data processing and data cleaning procedures employed in this study. (PDF)

S4 Text Compilation of R program code used in the various analytical steps in this study. (DOCX)

S1 Data Summary of birth rates among scientific journals analyzed in this study. (PDF)

S2 Data Summary of death rates among scientific journals analyzed in this study. (PDF)

S3 Data Summary of birth rates among scientific journals analyzed in this study, excluding OA journals. OA, open access.
(PDF)

S4 Data Summary of death rates among scientific journals analyzed in this study, excluding OA journals. OA, open access.
(PDF)

S5 Data Summary of journal birth and death rates, as summarized from the PubMed Central database. (PDF)

S1 Fig Summary of proportion of journals that are OA, by year. OA, open access.
(TIF)

S2 Fig Counts of numbers of journals active by year, separated by subject area, excluding OA journals. OA, open access.
(TIF)

S3 Fig Summary of journal birth rates by year, excluding OA journals. OA, open access.
(TIF)

S4 Fig Summary of journal death rates by year, excluding OA journals. OA, open access.
(TIF)

S5 Fig Summary of journal birth rates (smoothed) by year, excluding OA journals. OA, open access.
(TIF)

S6 Fig Summary of journal death rates (smoothed) by year, excluding OA journals. OA, open access.
(TIF)

S7 Fig Summary of journal birth and death rates by year, based on data from PubMed Central. (TIFF)
