# Supplementary material for: The NIH public access policy did not harm biomedical journals
Source: PLoS Biol. 2019 Oct 23;17(10):e3000352. doi: 10.1371/journal.pbio.3000352 (PMC6808382; doi:10.1371/journal.pbio.3000352)
Supplement: S1 Text — (DOCX) [file pbio.3000352.s001.docx]

**Online Content: Methods**

The methods used in the derivation of the analyses reported herein are described in full detail in S1 Protocols, including R scripts for many of the key steps involved. The data necessary for this analysis were drawn from Ulrich’sWeb Global Serials Directory. We defined a search that retrieved journal status (active, ceased, merged/incorporated, or suspended), the subject classification, serial type, content type, whether the journal was refereed or peer-reviewed, the year in which the journal was created, as well as a number of other bibliographic identifiers. The initial retrieval was quite large (784,756 journal records). After filtering duplicate listings and limiting the set to include “Academic / Scholarly” journals that are refereed or reviewed, published in the United States, and for which status is either Active or Ceased, we had 18,372 records representing 15,921 active journals and 2451 journals that have ceased publication. We further refined our search and inclusion of records via parsing the Ulrich’sWeb “subject” field into individual subject areas (note that a given journal may fall into multiple subjects): AGRICUL included agricultural science; BIOMED included medical sciences, pharmacy, psychology, public health, and health facilities and administration; NATSCI included biology, geography, earth science, conservation, environmental studies, fish and fisheries, forests and forestry, and paleontology; PHYSCI included chemistry, astronomy, math, physics, statistics, and meteorology; ENGTECH included aeronautics, computers, engineering, energy, technology, and library (i.e., information sciences); and SOCSCI included anthropology, social science, sociology, archaeology, political science, population studies, and social services and welfare. A total of 10,980 journals fell into one or more of these subject areas: 208 AGRICUL, 4480 BIOMED, 1923 NATSCI, 1105 PHYSCI, 1815 ENGTECH, and 1449 SOCSCI journals.

Information on the year of termination of publication (“death”) for journals that ceased publication was retrieved through a separate set of queries (see S1 Protocols), and the resulting information was merged with the original dataset. Using data management tools in the R package survival , the data were reshaped as a year-by-year “survival spell” data structure, from which we tabulated annual summaries for calendar years. Annual journal counts are illustrated in Fig 1, and birth and death rates for journals through time are provided in S2 Data.

The generalized additive model was estimated with routines provided by the R package mgcv [24]. The GAM uses a negative binomial distribution (with offset) to estimate the birth rate, which is then scaled to reflect births per 1000 journals in the previous year. The Cox proportional hazard model for journal closures was estimated using a procedure described by Whitehead [25] as described in the mgcv package’s documentation for the function cox.pht [24].
