# Supplementary material for: The NIH public access policy did not harm biomedical journals
Source: PLoS Biol. 2019 Oct 23;17(10):e3000352. doi: 10.1371/journal.pbio.3000352 (PMC6808382; doi:10.1371/journal.pbio.3000352)
Supplement: S2 Text — (PDF) [file pbio.3000352.s002.pdf]

Harvesting Journal Data from Ulrich

Ulrich provides two types of data: a list with the basic information for all journals, and a detailed web page for each journal. User cannot visit all of the records in the list directly, but can search the journals via a keyword, and explore the matched records page by page on Ulrich's website; inexplicably, no field in this list summarizes the end year of publication for each journal (endYear). EndYear can be found in the detailed web page for each journal.

We therefore used three steps to harvest data from the Ulrich's database, working via the Ulrich API. (1) We downloaded all records from the list via 36 individual queries, based on keywords of a* to z* and 0* to 9*, where "*" indicates any succeeding characters. (2) We downloaded the detailed web pages for all journals with status listed as "ceased," "merged / incorporated," or "suspended" (= potential journal death). Finally, (3) extracted endYear information from the detailed web pages.

Ulrich provides a user interface by which to visit all records in its database page by page. Users can explore them on Ulrich's website, but this site is quite hard to analyze in a structured way. We found that these web pages were rendered as a JSON object (= pure data). As such, he wrote an R script (download_all_records_from_Ulrich.r) by which to skip the rendering the process, and download the JSON objects directly. The API URI template is "http://ulrichsweb.serialssolutions.com/api/API-KEY/search?query=title:P1*&start=P2&rows=P3". P1 is the keyword for the query (see above: a to z, and 0 to 9). P2 is the starting row number, and P3 is the number of rows returned by a single query. This step yielded 784,756 records from Ulrich, including journals, books, and other sorts of publications.

Downloading the detailed web pages of the potential dead journals only (see above: serialTypes Journal and status ceased, merged / incorporated, or suspended. This procedure yielded 14,070 potentially "dead" journals. The script 'download_details.r' was used to download the detailed web pages for these journals from Ulrich. The URL template to download the page for each journal is http://ulrichsweb.serialssolutions.com/titleDetails/titleId?_=XXXX. In this template, 'titleId' should be replaced with the titleId of the journal, and XXXX is the session ID, which one obtains when visiting the Ulrich website from an authorized location. Webpages downloaded were stored as local files for the next analysis (see below). Finally, the script 'add_end_year.R' was to extract the endYear information from the locally stored, detailed web pages; this script add the endYear information to the basic Ulrich records.
