# Supplementary material for: The NIH public access policy did not harm biomedical journals
Source: PLoS Biol. 2019 Oct 23;17(10):e3000352. doi: 10.1371/journal.pbio.3000352 (PMC6808382; doi:10.1371/journal.pbio.3000352)
Supplement: S3 Text — (PDF) [file pbio.3000352.s003.pdf]

Technical Report: Importing the Ulrich's Web Data

Abstract

This is a report on procedures for data extraction and re-classifcation in the project entitled, "Did the NIH public access policy really kill biomedical journals?" by A. Townsend Peterson, Paul E. Johnson, Narayani Barve, Ada Emmett, Marc Greenerg, Josh Bolick and Huijie Qiao. It describes the procedures used to download, filter and classify journals by subject.

A companion R file that has the same name as this report, with the suffix ".R", is available.

1 Data Extraction

The records for publications were extracted by structured query language (SQL) requests on the *Ulrich's Web* applications programming interface (API).

The download process results in two files, "all_ulrich_data-20190415.rdata" and "filtered-20190416.rdata". The former is a comprehensive account of the records available from *Ulrich's Web*, which offers many serial publications, some of which have ceased publication long in the past. The variables in which we are most interested are the start year and the subject classification. The latter file includes the information about journals that have ceased publication. It has the year in which the journal was discontinued.

2 Data Import

There were 784756 rows of information in "all_ulrich_data-20190415.rdata".
In "filtered-20190416.rdata" there are 14070 rows.

3 Preliminary Row Filtering

There are some rows in each file that are redundant. A record is considered redundant if the following items are identical: titleId, title, startYear, status, formats. After removing duplicates, the row count drops from 784756 to 782289.

There are duplicated rows that remain because many journals are disseminated in more than one format. One example is *Particle Accelerators*, which appears in four rows in both of the data sets.

1425 Jayhawk Blvd.
Watson Library, Suite 470
Lawrence, KS 66045-7594

Web: https://crmda.ku.edu
Email: crmda@ku.edu
Phone: 785-864-3353

	titleId	title	startYear	status	formats	endYear
3571	39011	Particle Accelerators	1969	Ceased	Print	2000
3572	39012	Particle Accelerators		Ceased	CD-ROM	
3573	39013	Particle Accelerators		Ceased	Online	
3999	279099	Particle Accelerators		Ceased	Microform	

De-duplicating these entries is discussed below in section 5.2

4 Merge

Next we integrate the information for the journals that have been closed. This is a horizontal "join" operation. As a merge diagnostic, we find that all of the titles that appear in the second data set are matched in the first one. In other words, the merge is a success.

5 Filter Rows

5.1 Eliminate by publication type

We further limit our attention to publication content types that are classified by *Ulrich's* as "Academic / Scholarly", have serialTypes equal to "Journal", and are published in the United States.

We began this phase with 782289 rows of journal information. Limiting our attention to the content type "Academic / Scholarly" reduced that to 197216. The requirement that the country be "United States" reduced the number of journal rows to 45467. And by excluding any serial types that are not "Journal", we arrive at 34787 rows.

5.2 Duplicate Titles

Now we confront the problem that some titles in the collection are represented by several rows. This occurs because a journal may begin with a print version, which is later accompanied by (or replaced by) an online or microfilm version. In our analysis, we do not want to treat all of these as separate journals. The total number of unique values for journal title is 18835 and among them there are 11653 journal titles with more than one row of information.

As a litmus test, we will monitor the journal *Zygon*. From the outset, we have:

	titleId	title	startYear	formats	endYear
737212	55647	Zygon	1966	Print	
781202	55648	Zygon	1997	Online	
781964	284938	Zygon		Microform	

The corrected data for *Zygon* should have one row, with `startYear` 1966 and `endYear` NA.

The data reduction method is to split the data by row blocks, one for each observed title. Then sift through the rows to find out if there is information in them that is unique. We created a function called `delteuninformativerows` that can look at the repeated lines and eliminate the ones that contribute no information. This is available in the R code companion file.

Using a filtering algorithm, we reduce the number of data rows to 18835. The de-duplication doublecheck indicates we were successful, there are no more titles with more than one row. For example, the result for *Zygon* is

	title	startYear	formats	endYear
18834	Zygon	1966	Print	

5.3 Unsolved duplicate title problems

In the previous effort, we concentrated on eliminating repeated titles. That meant titles that were exactly the same. We still have a second kind of duplication. There are journals with multiple rows that should be compressed into 1 row, such as:

1. The Journal of Musculoskeletal Medicine (Online)
2. The Journal of Musculoskeletal Medicine (Print)

or

1. The Journal of Contemporary Health Law and Policy (Online)
2. The Journal of Contemporary Health Law and Policy (Print)

There are additional complications where the startYears for the two versions of the journals differ. Clearly, we want to keep the first startYear value, and the latest endYear. One (among many) interesting examples is the journal *Northeastern Naturalist*:

	title	status	startYear	endYear
13211	Northeastern Naturalist (Online)	Active	1993	
13212	Northeastern Naturalist (Print)	Ceased	199?	1993

There are two obvious problems. First, there are two rows where we want one. Second, the start year includes a question mark. If any of the records for a journal has a valid year for the start or end year, we will use that information.

Northeastern Naturalist (NN) started in 1993 as a print journal, but after a brief while, it became an online journal only. As a result, we combine the rows and treat the starting year as 1993.

There are 385 journals that have titles that are duplicated, except for the inclusion of the word “(Print)” and “(Online)” in the titles. We filter those by requiring the publisher and the subject of the journals must be identical.

Using a de-duplication strategy (which can be inspected in the accompanying R file), we have reduced the number of rows to 18372. The preliminary spot check on three of the journals mentioned above is encouraging:

	status	startYear	endYear
Northeastern Naturalist	Active	1993	
The Journal of Contemporary Health Law and Policy	Active	1984	
The Journal of Musculoskeletal Medicine	Active	1983	

6 Assign Subject Classifications

The *Ulrich's Web* system uses a multiple keword classification system. In this section, we discuss our procedure for sorting through the classification provided by *Ulrich's Web*. We create both a "primary" subject classification for each journal, as well as indicators for each journal indicating if it might belong to the families defined by agriculture, bio-medical research, engineering, social science, natural science, or physical science.

6.1 Subject data format

The subject listings in the *Ulrich's Web* data base are multiple-topic indicators separated by punctuation, commas, colons, and dashes. We have exerted great care to parse the punctuation correctly.

Consider the *American Journal of Materials Science* (*AJMS*), for which the subject string is

```
CERAMICS, GLASS AND POTTERY,ENGINEERING - CHEMICAL ENGINEERING,ENGINEERING - ENGINEERING
    MECHANICS AND MATERIALS,METALLURGY
```

This case is useful as an illustration of the complicated format of the subject information. The subject string uses commas both as a separator for large items and also for punctuation within the items. The commas that are not followed by white space are terminators for subjects. The AJMS subject heading should be seen as

1. CERAMICS, GLASS AND POTTERY
2. ENGINEERING - CHEMICAL ENGINEERING
3. ENGINEERING - ENGINEERING MECHANICS AND MATERIALS
4. METALLURGY

Another interesting case is the *Journal of Political Economy* (*JOPE*), for which the subject string is

```
BUSINESS AND ECONOMICS,POLITICAL SCIENCE
```

That subject string should be understood as

1. BUSINESS AND ECONOMICS
2. POLITICAL SCIENCE

The primary subject identifiers are categorized into larger sets by a matching system using a taxonomy that was designed by our subject matter experts.

subject.areas	keywords
BIOMED	MEDICAL SCIENCES, PHARMACY AND PHARMACOLOGY, PSYCHOLOGY, PUBLIC HEALTH AND SAFETY, HEALTH FACILITIES AND ADMINISTRATION
NATSCI	BIOLOGY, GEOGRAPHY, EARTH SCIENCES, ENVIRONMENTAL STUDIES, FISH AND FISHERIES, FORESTS AND FORESTRY, PALEONTOLOGY, CONSERVATION
PHYSCI	CHEMISTRY, ASTRONOMY, MATHEMATICS, PHYSICS, STATISTICS, METEORLOGY
ENGTECH	AERONAUTICS AND SPACE FLIGHT, COMPUTERS, ENGINEERING, ENERGY, TECHNOLOGY, LIBRARY AND INFORMATION SCIENCES
SOCSCI	ANTHROPOLOGY, SOCIAL SCIENCES, SOCIOLOGY, ARCHAEOLOGY, POLITICAL SCIENCE, POPULATION STUDIES, SOCIAL SERVICES AND WELFARE
AGRICUL	AGRICULTURE

6.2 Our Classification Strategy

We check each of the separate elements in the *Ulrich's* subject data. In the case of the *AJMS*, the original subject classification is reported as:

```
[1] "CERAMICS, GLASS AND POTTERY,ENGINEERING - CHEMICAL ENGINEERING,ENGINEERING -
    ENGINEERING MECHANICS AND MATERIALS,METALLURGY"
[2] "BUSINESS AND ECONOMICS,POLITICAL SCIENCE"
```

First, we parse the subject string at the comma that is not followed by a space, so that the subject for the AJMS is seen as 4 elements.

The detailed specifier after the long dash is then stripped. The remaining elements are matched against our subjects.

```
[1] "CERAMICS, GLASS AND POTTERY"
[2] "ENGINEERING - CHEMICAL ENGINEERING"
[3] "ENGINEERING - ENGINEERING MECHANICS AND MATERIALS"
[4] "METALLURGY"
```

```
[1] "CERAMICS, GLASS AND POTTERY" "ENGINEERING"
[3] "ENGINEERING"                 "METALLURGY"
```

We classify journals from "left to right." If there is a match in the first term, we create a classification. When the first element in the subject is not matched, then the subsequent elements can be considered.

We create a matrix in which the integer values represent which terms are matched. Consider a few rows:

	BIOMED	NATSCI	PHYSCI	ENGTECH	SOCSCI	AGRICUL
164399	1					
800201	2			3		
592789			2	1		
737879				2		
48302					2	

The rows represent journals, the columns represent classification. If there is a 1 in a cell, it means that the first term in the subject vector was matched by a keyword within our classification table. A 2 indicates that the second word in the *Ulrich's* classification matched one of our topics.

Following this procedure, we end up with individual indicator variables, one for each subject area (coded 0 or 1), as well as a "primary" subject indicator, which is the matching category which has the lowest score in the match matrix. If the whole row is missing–filled with NA values–then no subject grouping is assigned.

The *American Journal of Materials Science* is the second-to-last line in the matrix displayed above. There is no first term match, but the second term is matched by an engineering term. Hence, we classify AMJS as an ENGTECH journal.

The *Journal of Political Economy* is the last line in the matrix. Note that it has no first term matches, but the second subject topic term is matched by a social science keyword. We classify this as a SOCSCI journal.

Using this method, the number of journals that falls into the designated catetories is as follows:

	Count
AGRICUL	208
BIOMED	4480
ENGTECH	1815
NATSCI	1923
PHYSCI	1105
SOCSCI	1449
NA.	7392
Sum	18372

The Sum is greater than the sample size because some journals are classified in more than one subject area.

7 Creating Indicators to Facilitate Analysis

7.1 A marker for 'refereed' and 'reviewed' journals

The data has two classifier variables, "refereed" and "reviewed", which are distilled into one indicator in our data collection. The tabulation is as follows, for each subject grouping.

	AGRICUL	BIOMED	ENGTECH	NATSCI	PHYSCI	SOCSCI	NA
refereed	113	3056	1051	1138	680	593	2975
reviewed	3	25	39	22	16	61	359
both	30	417	259	285	185	365	1230
neither	62	982	466	478	224	430	2828

If we exclude the journals that are neither refereed nor reviewed, we reduce the number of subject-classified journals by 2642 journals.

8 A marker for status: Active or Ceased

The journal's status can be scored with a number of labels. Almost all journals are Active or Ceased, as we see in the following:.

	Count
Active	14831
Announced Never Published	174
Ceased	2451
Forthcoming	30
Merged / Incorporated	181
Researched / Unresolved	648
Suspended	57

We exclude from consideration the journals that have status "Announced Never Published", "Forthcoming", and so forth. We create an indicator variable `statusf` which is missing for all status values except Active and Ceased.

	Active	Ceased	NA
Active	14831	0	0
Announced Never Published	0	0	174
Ceased	0	2451	0
Forthcoming	0	0	30
Merged / Incorporated	0	0	181
Researched / Unresolved	0	0	648
Suspended	0	0	57

9 Start and End year for journals

In order to conduct a survival analysis, we need the start years for all journals under consideration. We also need the termination year for all of the Ceased journals. In the startYear summary, the first value is blank and there are several years including "?". Those cases are omitted from the analysis, but for record keeping we retain both the original information about the start and end year as well as the cleaned version. We end up with 2 sets of year variables,

1. startYear: original data, includes blanks and "?" values from input data
2. startYear2: all non-integer values converted to missing values
3. endYear: original data, includes blanks and "?" values from input data
4. endYear2: endYear + 1, all non-integer values converted to missing values

In the data for journal termination date, there is an obvious typographical error indicating that "NHSA Dialog" was in 3012. That value should be 2012.

There are some journals for which the `startYear` and `endYear` are identical. That happens because the variable `endYear` is the last year of publication, rather then the year after termination. Hence, for calculating a journal's age (or duration of survival), we use $endYear + 1$.

The variable "duration" will represent the number of years that a journal existed before closure. For journals that have not closed, the "duration" is the difference between the maximum value of the observed end year plus 1 and the journal's start year.

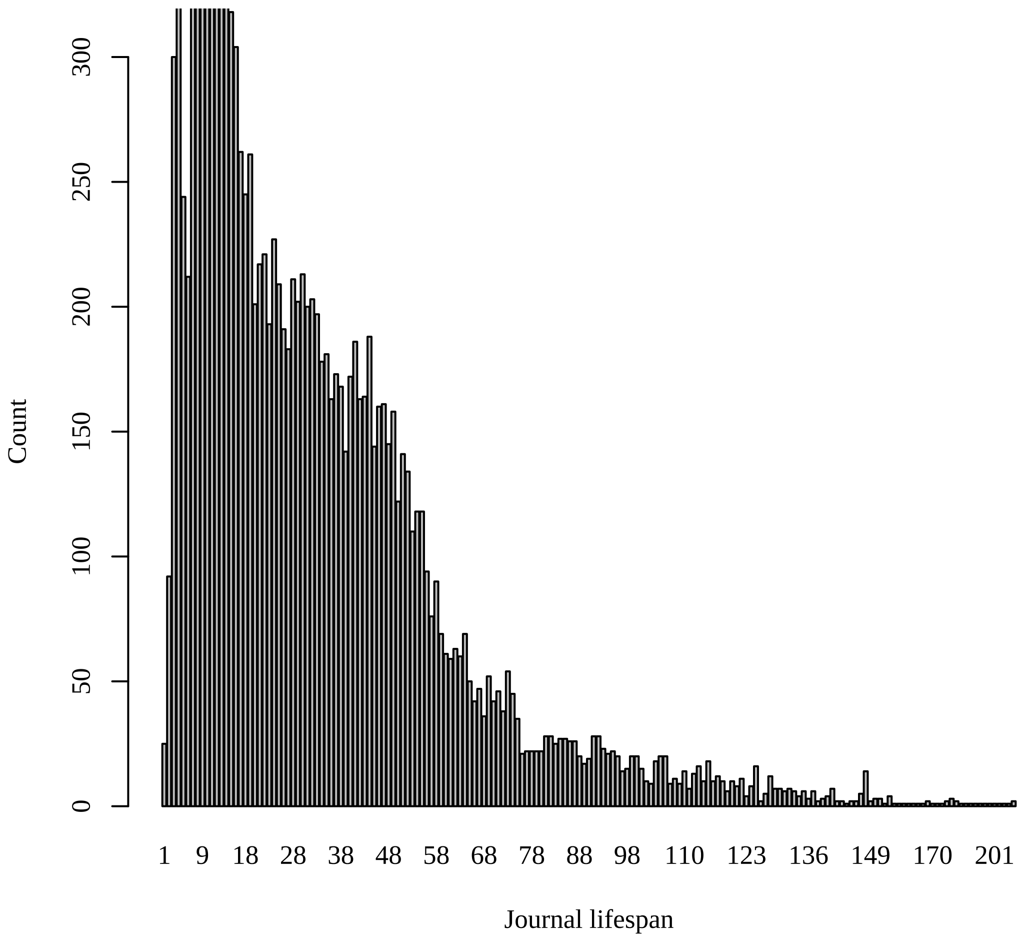

9.1 Exclude Very Old Journals

The oldest journal in our collection began in 1758. We have limit our consideration to journals that were created before 1900. Doing so removes from consideration 222 journals.

9.2 Drop journals that closed before 1980

There are a handful of journals that have end years before 1980. We exclude them from further consideration. The omission of journals that closed before 1980 affected 14 rows of information.

9.3 Variables for Duration Analysis: failvar, cohort

For survival analysis, we need a variable indicating whether a journal has ceased to exist before the end of the period under consideration. This new variable, `failvar`, is coded 0 if no "event" (closure) occurred, and 1 if a closure event occurred.

The failure variable is coded as missing for all of the status values except Active or Ceased.

	0	1	NA
Active	14628	0	0
Announced Never Published	0	0	174
Ceased	0	2425	0
Forthcoming	0	0	30
Merged / Incorporated	0	0	181
Researched / Unresolved	0	0	642
Suspended	0	0	56

We have experimented with various cohort groupings for the journals. The primary investigation isolated journals that existed at or after 1980. As a result, the ones that were created before, say, 1970, are not a representative sample of all journals. The ones created after 1980, however, are more likely to be representative. As a result, it seems wise to isolate journals into several date-of-birth cohorts so that we can track their survival separately.

The variable `cohort` is the decade-based tabulation

One striking fact is apparent from the cross tabulation of the new cohorts with the status indicator: There has been a prolific expansion in the number of journals since 2000.

bf1970	1970s	1980s	1990s	2000s	2010s	NA	Sum
2306.00	1471.00	1613.00	1834.00	2845.00	4043.00	516.00	14628.00
270.00	258.00	393.00	515.00	466.00	417.00	106.00	2425.00
113.00	117.00	158.00	187.00	200.00	26.00	282.00	1083.00
2689.00	1846.00	2164.00	2536.00	3511.00	4486.00	904.00	18136.00

One point of concern is that the data on the last year of publiction for 542 of the journals that have ceased publication.

10 Exported Data snapshots

The process of filtering and reclassifying that is described in this report generates working data files in both comma separated variable (CSV) and R data serialization (RDS) formats (jrnls4.{csv,rds}).
