# Supplementary material for: The NIH public access policy did not harm biomedical journals
Source: PLoS Biol. 2019 Oct 23;17(10):e3000352. doi: 10.1371/journal.pbio.3000352 (PMC6808382; doi:10.1371/journal.pbio.3000352)
Supplement: S4 Text — (DOCX) [file pbio.3000352.s004.docx]

**download_all_records_from_Ulrich.R**

#-------------------------------------------------------

#Download the records from Ulrich

#------------------------------------------------

library("rjson")

library("data.table")

setwd("~/ulrich20190407")

args = commandArgs(trailingOnly=TRUE)

#Set up the start letter for the query request. a to z and 0 to 9

start_letter<-args[1]

#basic url to download the record from Ulrich

url<-"http://ulrichsweb.serialssolutions.com/api"

#the api key, provided by KU

api_key<-"XXXXXX"

#set up the lines to download per request/page.

rows<-50

# template of the request URL

json_url_template <- "%s/%s/search?query=title:%s*&start=%d&rows=%d"

# set up the folder to save the records

base_folder<-"~/ulrich20190407/data/%s"

if (dir.exists(sprintf(base_folder, start_letter))){

next()

}

json_file_template<-"%s/title_%s.start_%d.rows_%d.RData"

#start to download the Ulrich data page by page.

json<-rjson::fromJSON(file=sprintf(json_url_template, url, api_key, start_letter, 1, rows))

dir.create(sprintf(base_folder, start_letter))

#save the downloaded records (in JSON format) to a local file

save(json, file=sprintf(json_file_template, sprintf(base_folder, start_letter), start_letter, 1, rows))

#traversing all the pages to download all the records from Ulrich.

if (json[1]$status=="Success"){

totalRecords<-json$totalRecords

current_start<-rows+1

while(current_start<totalRecords){

saved_file<-sprintf(json_file_template, sprintf(base_folder, start_letter),

start_letter, current_start, rows)

print(paste(current_start, totalRecords, sep=" / "))

url_item<-sprintf(json_url_template, url, api_key, start_letter, current_start, rows)

print(url_item)

if (!file.exists(saved_file)){

json<-rjson::fromJSON(file=url_item)

save(json, file=saved_file)

}

current_start<-current_start+rows

}

}

**download_details.R**

#-------------------------------------------------------

# Because the Ulrich table has no endYear information,

# we need to download the detail page for each journal,

# and extract the endYear from it.

# This script is used to finish the task above.

#------------------------------------------------

setwd("~/Dropbox/Papers/OA/Plos Biology/Script/Ulrich")

# load the Ulrich records

load("data/filtered-20190416.rdata")

df_filter$endYear<-NA

dim(df_filter)

# filter the potential dead journals via the status

df_filter<-df_filter[which((df_filter$status=="Ceased")|

(df_filter$status=="Merged / Incorporated")|

(df_filter$status=="Suspended")),]

library("RCurl")

# the URL template to download the detail page for each journal.

# %s is the titleId of the journal, and XXXX is the session ID, which

# can get when a user visits Ulrich website from an authorized location (KU).

url<-"http://ulrichsweb.serialssolutions.com/titleDetails/%s?_=XXXX"

i<-1

# download the detail pages, and save them to local files.

for (i in c(1:nrow(df_filter))){

content_url<-sprintf(url, df_filter[i,]$titleId)

result_file<-sprintf("data_details/%s.html", df_filter[i,]$titleId)

if (file.exists(result_file)){

next

}

write.table("", result_file)

print(content_url)

content<-getURL(sprintf(url, df_filter[i,]$titleId))

write.table(content, result_file)

}

**dataimport-201904.R**

### R code from vignette source 'dataimport-201904-uniquebackupstring201904231822.Rnw'

### Encoding: UTF-8

###################################################

### code chunk number 1: dataimport-201904-uniquebackupstring201904231822.Rnw:21-22

###################################################

if(exists(".orig.enc")) options(encoding = .orig.enc)

###################################################

### code chunk number 2: tmpout

###################################################

if(!dir.exists("tmpout")) dir.create("tmpout", showWarnings=FALSE)

###################################################

### code chunk number 3: excludemeRoptions

###################################################

if(!dir.exists("tmpout"))dir.create("tmpout", recursive = TRUE)

opts.orig <- options()

options(device = pdf)

options(width=70, prompt="> ")

options(useFancyQuotes = FALSE)

set.seed(12345)

par.orig <- par(no.readonly=TRUE)

pjmar <- c(5.1, 5.1, 1.5, 2.1)

options(SweaveHooks=list(fig=function() par(mar=pjmar, ps=10)))

pdf.options(onefile=FALSE, family="Times", pointsize=10)

###################################################

### code chunk number 4: setup

###################################################

ddir <- "../../data"

wdir <- "../../workingdata"

odir <- "output"

if(!file.exists(odir)) dir.create(odir)

fn1 <- "all_ulrich_data-20190415.rdata"

fn2 <- "filtered-20190416.rdata"

#fn2 <- "filtered_all_journals_20190417.RData"

today <- format(Sys.time(), "%Y%m%d")

fn1.tex <- "all\\\\_ulrich\\\\_data-20190415.rdata"

fn2.tex <- "filtered-20190416.rdata"

##fn2.tex <- "filtered\\\\_all\\\\_journals\\\\_20190417.RData"

###################################################

### code chunk number 5: alldata

###################################################

##jrnls.0 <- readRDS(file.path(ddir, fn1))

load(file.path(ddir, fn1))

jrnls.0 <- df

j0 <- dim(jrnls.0)

###################################################

### code chunk number 6: endyeardata1

###################################################

# jrnls_endyear <- readRDS(file.path(ddir, fn2))

load(file.path(ddir, fn2))

#jrnls_endyear <- df_filter_all_journal

jrnls_endyear <- df_filter

j1 <- dim(jrnls_endyear)

## key identifiers, called matchvars.

matchvars <- c("titleId", "title", "startYear", "status", "formats")

## Keep only the variables we need from jrnls_endyear

keepers <- c(matchvars, "endYear")

jrnls_endyear <- jrnls_endyear[ , keepers]

###################################################

### code chunk number 7: dataimport-201904-uniquebackupstring201904231822.Rnw:165-167

###################################################

## Did not make latex work here, though

library(xtable)

###################################################

### code chunk number 8: jrnlsrowfilter1

###################################################

jrnls_dupes <- duplicated(jrnls.0[ , matchvars])

jrnls.0 <- jrnls.0[!jrnls_dupes, ]

j2 <- dim(jrnls.0)

###################################################

### code chunk number 9: endyeardata2

###################################################

## There are no duplicated rows on the match vars:

jrnls_endyear_dupes <- duplicated(jrnls_endyear[ , matchvars])

table(jrnls_endyear_dupes, exclude = NULL)

###################################################

### code chunk number 10: endyeardata3

###################################################

j99 <- jrnls_endyear[jrnls_endyear$title == "Particle Accelerators", ]

print(xtable(j99), floating=FALSE)

###################################################

### code chunk number 11: merge1

###################################################

## Add markers to facilitate diagnostics on unmatched cases

jrnls.0$from.x = TRUE

jrnls_endyear$from.y <- TRUE

jrnls <- merge(jrnls.0, jrnls_endyear,

by = matchvars,

sort = FALSE, all.x = TRUE, all.y = TRUE)

###################################################

### code chunk number 12: merge1a

###################################################

j3 <- dim(jrnls)

###################################################

### code chunk number 13: merge2

###################################################

## merge diagnostic:

t1 <- table("from Ulrich" = jrnls$from.x, "from endYear data" = jrnls$from.y, exclude = NULL)

t1xt <- xtable(t1)

print(t1xt, floating=FALSE)

###################################################

### code chunk number 14: jrnlsrowfilter2

###################################################

# Restrict to US, Academic/Scholarly journals

jrnls <- jrnls[jrnls$contentTypes == "Academic / Scholarly", ]

j4 <- dim(jrnls)

jrnls <- jrnls[jrnls$country == "United States", ]

j5 <- dim(jrnls)

jrnls <- jrnls[jrnls$serialTypes == "Journal", ]

j6 <- dim(jrnls)

###################################################

### code chunk number 15: dupecheck1

###################################################

dupecheck <- duplicated(jrnls[ , c("title")])

table(dupecheck, exclude = NULL)

titledupes <- jrnls[dupecheck, "title"]

jrnls_dupes <- jrnls[jrnls$title %in% titledupes, ]

jrnls_dupes <- jrnls_dupes[order(jrnls_dupes$title, jrnls_dupes$formats), ]

(nofdupes <- length(unique(jrnls_dupes$title)))

## The first 50 repeated title lines

head(jrnls_dupes$title, 50)

## see them interactively

##View(head(jrnls_dupes, 200))

uniquetitles <- length(unique(jrnls$title))

###################################################

### code chunk number 16: zygonexample

###################################################

jrnlbytitle <- split(jrnls, f = list(jrnls$title))

j99 <- jrnlbytitle[["Zygon"]][ , c("titleId", "title", "startYear", "formats", "endYear")]

print(xtable(j99), floating=FALSE)

###################################################

### code chunk number 17: dupefix1

###################################################

## This function will receive a block and then delete

## repeated rows, using various kinds of guessing

## that are explained within

deleteuninformativerows <- function(X){

if (NROW(X) == 1) return(X)

## Find rows where both startYear and endYear are uninformative, and

## delete those rows as long at it leaves at least 1 good row

X$removenow <- ifelse(X$startYear %in% c("", NA) & X$endYear %in% c("", NA), TRUE, FALSE)

if (NROW(X) - sum(X$removenow) >= 1){

X <- X[!X$removenow, , drop = FALSE]

} else {

## It wants to delete all of the rows, but only delete N - 1

ntoremove <- sum(X$removenow)

## remove rows 1 to ntoremove - 1

X <- X[-seq(1, ntoremove -1), , drop = FALSE]

}

if (NROW(X) == 1) {

X[ , "removenow"] <- NULL

return(X)

}

## That killed off the empty "online" and "microform" rows.

##

## Now get rid of rows that have startYear like "19??";

## If has question mark or "", startYearn will become NA

X$startYearn <- as.numeric(X$startYear)

X$removenow <- ifelse(X$startYearn %in% c("", NA) & X$endYear %in% c("", NA), TRUE, FALSE)

## If removal does not create a 0 row journal do it

if (NROW(X) - sum(X$removenow) >= 1){

X <- X[!X$removenow, , drop = FALSE]

} else {

## It wants to delete all of the rows, but only delete N - 1

ntoremove <- sum(X$removenow)

## remove rows 1 to ntoremove - 1

X <- X[-seq(1, ntoremove -1), , drop = FALSE]

}

if (NROW(X) == 1){

X[ , c("startYearn", "removenow")] <- NULL

return(X)

}

## Still have multiple rows, so keep only one with smallest

## startYear. Only do this if endYear is uninformative

## If startYear is not all NA

if(any(!is.na(X$startYearn))){

startLowest <- which.min(X$startYearn)

if (length(startLowest) == 1 && length(unique(X$endYear) < 2)){

X <- X[startLowest, , drop = FALSE]

}

if (NROW(X) == 1){

X[ , c("startYearn", "removenow")] <- NULL

return(X)

}

}

## Some cases have 2 rows with contradictory startYear information

## in a format like c("19??", "") or c("198?", "19??").

## This arises in 6 journal

## cases, in all of which startYear is "" or has a "?". If

## there are different values, we don't care much because

## unknown startYear will cause exclusion later. So just keep

## one row.

if(length(unique(X$startYear)) > 1 & all(is.na(as.numeric(X$startYear)))){

X <- X[1, ]

}

## Previous eliminates all but 2 sets of repeats. These

## journals have identical startYear == 19?? and given endYear.

## if rows have same info, keep first one.

if(length(unique(X$startYear)) == 1 & length(unique(X$endYear)) == 1){

X <- X[1, ]

}

X[ , c("startYearn", "removenow")] <- NULL

X

}

###################################################

### code chunk number 18: dupefix2

###################################################

xxx <- lapply(jrnlbytitle, deleteuninformativerows)

## Warnings are harmless, they concern "19??" turning to NA by

## as.numeric()

jrnls2 <- do.call("rbind", xxx)

## The row names become a distraction

rownames(jrnls2) <- NULL

j7 <- dim(jrnls2)

###################################################

### code chunk number 19: dupecheck2

###################################################

## This process required several cycles to clean

## the logic in the function above.

dupecheck <- duplicated(jrnls2[ , c("title")])

titledupes <- jrnls2[dupecheck, "title"]

if(length(titledupes) > 0){

jrnls2_dupes <- jrnls2[jrnls2$title %in% titledupes, ]

jrnls2_dupes <- jrnls2_dupes[order(jrnls2_dupes$title, jrnls2_dupes$formats), ]

## If jrnls_dupes has any rows, then we need to go check

print("FAILURE: There are repeated journal titles:")

jrnls2_dupes$title

}

###################################################

### code chunk number 20: zygon2

###################################################

t10 <- jrnls2[jrnls2$title == "Zygon", c("title", "startYear", "formats", "endYear")]

t10xt <- xtable(t10)

print(t10xt, floating=FALSE)

###################################################

### code chunk number 21: mordupe1

###################################################

j99 <- jrnls2[grep("Northeastern Naturalist" , jrnls2$title), c("title", "status", "startYear", "endYear")]

print(xtable(j99), floating=FALSE)

###################################################

### code chunk number 22: mordupe3

###################################################

## Shadi's suggestion

## jrnls2$title.chopped <- trimws(gsub("\\(.*\\)", "", jrnls2$title))

## dupedtitles <- jrnls2$title.chopped[duplicated(jrnls2$title.chopped)]

jrnls2$title.chopped <- trimws(gsub("[:(:]Print[:):]|[:(:]Online[:):]", "", jrnls2$title))

dupedtitles <- jrnls2$title.chopped[duplicated(jrnls2[ , c("title.chopped", "publisher", "subject")])]

dupecount <- length(unique(dupedtitles))

jrnls2$titleisdupe <- jrnls2$title.chopped %in% dupedtitles

table(jrnls2$titleisdupe)

###################################################

### code chunk number 23: dataimport-201904-uniquebackupstring201904231822.Rnw:488-490

###################################################

## Make inspection easier by sorting

jrnls2 <- jrnls2[order(jrnls2$title.chopped), ]

###################################################

### code chunk number 24: mordupe4

###################################################

jrnls2.split <- split(jrnls2, f = jrnls2$title.chopped)

###################################################

### code chunk number 25: mordupe5

###################################################

xxx <- lapply(jrnls2.split, deleteuninformativerows)

## Warnings are harmless, they concern "19??" turning to NA by

## as.numeric()

jrnls3 <- do.call("rbind", xxx)

j8 <- dim(jrnls3)

###################################################

### code chunk number 26: mordupe6

###################################################

k1 <- jrnls3[grep("Northeastern Naturalist" , jrnls3$title), c("status", "startYear", "endYear")]

k2 <- jrnls3[grep("The Journal of Contemporary Health Law and Policy" , jrnls3$title), c("status", "startYear", "endYear")]

k3 <- jrnls3[grep("The Journal of Musculoskeletal Medicine" , jrnls3$title), c("status", "startYear", "endYear")]

kk <- rbind(k1, k2, k3)

print(xtable(kk), floating=FALSE)

###################################################

### code chunk number 27: cleanup10

###################################################

jrnls3[ , c("title.chopped", "titleisdupe")] <- NULL

jrnls3[ , c("from.x", "from.y")] <- NULL

###################################################

### code chunk number 28: mordupe10 (eval = FALSE)

###################################################

## ## How I conducted visual inspection of titles that seem to be

## ## same, except for parentheses. This shows very few duplicated

## ## journals remain

## jrnls3t <- jrnls3

## jrnls3t$title.chopped <- trimws(gsub("\\(.*\\)", "", jrnls3t$title))

## dupedtitles <- jrnls3t$title.chopped[duplicated(jrnls3t$title.chopped)]

## (dupecount <- length(unique(dupedtitles)))

## jrnls3t$titleisdupe <- jrnls3t$title.chopped %in% dupedtitles

## if(interactive()) View(jrnls3t[jrnls3t$titleisdupe , c("title", "title.chopped", "publisher")])

## rm(jrnls3t)

###################################################

### code chunk number 29: ajms

###################################################

ajms.subj <- jrnls2[jrnls2$title == "American Journal of Materials Science", "subject"]

###################################################

### code chunk number 30: ajmssubj2

###################################################

cat(ajms.subj)

###################################################

### code chunk number 31: jope1

###################################################

jope.subj <- jrnls2[grep("^Journal of Political Economy", jrnls2$title), "subject"]

###################################################

### code chunk number 32: jope2

###################################################

cat(jope.subj)

###################################################

### code chunk number 33: trackers

###################################################

trackme <- c("Journal of Political Economy", "American Journal of Materials Science")

trackmeid <- c("737879", "48302")

###################################################

### code chunk number 34: subgen

###################################################

subtypes <-

list(BIOMED = c('MEDICAL SCIENCES', 'PHARMACY AND PHARMACOLOGY',

'PSYCHOLOGY', 'PUBLIC HEALTH AND SAFETY',

'HEALTH FACILITIES AND ADMINISTRATION'),

NATSCI = c('BIOLOGY', 'GEOGRAPHY',

'EARTH SCIENCES', 'ENVIRONMENTAL STUDIES',

'FISH AND FISHERIES', 'FORESTS AND FORESTRY',

'PALEONTOLOGY', 'CONSERVATION'),

PHYSCI = c('CHEMISTRY', 'ASTRONOMY', 'MATHEMATICS', 'PHYSICS',

'STATISTICS', 'METEORLOGY'),

ENGTECH = c('AERONAUTICS AND SPACE FLIGHT', 'COMPUTERS',

'ENGINEERING', 'ENERGY', 'TECHNOLOGY',

'LIBRARY AND INFORMATION SCIENCES'),

SOCSCI = c('ANTHROPOLOGY', 'SOCIAL SCIENCES', 'SOCIOLOGY',

'ARCHAEOLOGY', 'POLITICAL SCIENCE',

'POPULATION STUDIES', 'SOCIAL SERVICES AND WELFARE'),

AGRICUL = c('AGRICULTURE'))

###################################################

### code chunk number 35: subgenbeauty

###################################################

keywords = sapply(subtypes, paste, collapse = ", ")

fakedf <- data.frame("subject.areas" = names(keywords),

keywords = keywords)

fakedfxt <- xtable(fakedf, align = c("l", "l", "p{5in}"))

print(fakedfxt, include.rownames=F, floating=FALSE)

###################################################

### code chunk number 36: dataimport-201904-uniquebackupstring201904231822.Rnw:647-648

###################################################

jrnls3[jrnls3$title %in% trackme , c("subject")]

###################################################

### code chunk number 37: newsep1

###################################################

## Insert a SEParator where we think it should be

subjnew1 <- gsub("(,(?=\\S))", "__SEP__", jrnls3$subject, perl = TRUE)

subjnewsplit <- strsplit(subjnew1, split = "__SEP__", fixed = TRUE)

## Use titleId as names

names(subjnewsplit) <- jrnls3$titleId

###################################################

### code chunk number 38: newsep2

###################################################

subjnewsplit[["737879"]]

###################################################

### code chunk number 39: newsep3

###################################################

subjnewsplit2 <- lapply(subjnewsplit, function(x) trimws(gsub("[:|-].*", "", x)))

names(subjnewsplit2) <- names(subjnewsplit)

subjnewsplit2[["737879"]]

###################################################

### code chunk number 40: newsep4

###################################################

matches <- matrix(NA, ncol = length(subtypes), nrow = length(subjnewsplit2),

dimnames = list(names(subjnewsplit2), names(subtypes)))

for(i in names(subtypes)){

#browser()

withbars <- paste0(subtypes[[i]], collapse = "|")

reslt <- vapply(subjnewsplit2, function(x){

itemmatch <- grep(withbars, x, perl=TRUE, ignore.case=TRUE)

itemmatch <- if(length(itemmatch) == 0) NA else min(itemmatch)

}, integer(1)

)

matches[ ,i] <- reslt

}

###################################################

### code chunk number 41: newsep5

###################################################

m1 <- matches[c("164399", "800201", "592789", trackmeid), ]

m1xt <- xtable(m1)

print(m1xt, floating=FALSE)

###################################################

### code chunk number 42: newsep6

###################################################

jrnls3$subject.grouped2 <- apply(matches, 1, function(x){

if(all(is.na(x))) NA else colnames(matches)[which.min(x)]

})

jrnls3$subject.grouped2 <- toupper(jrnls3$subject.grouped2)

###################################################

### code chunk number 43: newsep8

###################################################

t5 <- addmargins(table("Count" = jrnls3$subject.grouped2, exclude = NULL))

# t5[-c(7,8), 7]

t5xt <- xtable(t5, digits=0)

print(t5xt, floating = FALSE)

###################################################

### code chunk number 44: dataimport-201904-uniquebackupstring201904231822.Rnw:751-755

###################################################

rownames(jrnls3) <- jrnls3$titleId

matches[is.na(matches)] <- 0

matches[matches > 1] <- 1

jrnls3 <- merge(jrnls3, matches, by = "row.names", all=TRUE)

###################################################

### code chunk number 45: newsep9

###################################################

## group classification snapshot file

keepers <- c("titleId", "title", "subject", "AGRICUL", "BIOMED", "ENGTECH", "NATSCI", "PHYSCI", "SOCSCI")

ofn <- paste0("jrnls-subjects-", today, ".csv")

library(kutils)

file.backup(file.path(odir, ofn))

write.csv(jrnls3[ , keepers], file = file.path(odir, ofn), row.names = FALSE)

###################################################

### code chunk number 46: refereedfilter

###################################################

jrnls3$rrev <- factor(ifelse(jrnls3$refereed == "TRUE" & jrnls3$reviewed == "TRUE", "both",

ifelse(jrnls3$refereed == "TRUE", "refereed",

ifelse(jrnls3$reviewed == "TRUE", "reviewed",

"neither"))), levels = c("refereed", "reviewed", "both", "neither"))

###################################################

### code chunk number 47: refereedfilter2

###################################################

t3 <- table("refeered" = jrnls3$rrev, "single-subject grouping" = jrnls3$subject.grouped2, exclude = NULL)

t3xt <- xtable(t3)

print(xtable(t3xt), floating=FALSE)

###################################################

### code chunk number 48: dataimport-201904-uniquebackupstring201904231822.Rnw:810-812

###################################################

t1 <- table("Count" = jrnls3$status)

print(xtable(t1), floating = FALSE)

###################################################

### code chunk number 49: dataimport-201904-uniquebackupstring201904231822.Rnw:820-823

###################################################

jrnls3$statusf <- factor(jrnls3$status, levels = c("Active", "Ceased"))

t1 <- table("status original" = jrnls3$status, "status new version" = jrnls3$statusf, exclude = NULL)

print(xtable(t1), floating = FALSE)

###################################################

### code chunk number 50: createyearvars

###################################################

## Years with non-nonnumeric values are set as missings

jrnls3$startYear2 <- as.integer(jrnls3$startYear)

jrnls3$endYear2 <- as.integer(jrnls3$endYear)

###################################################

### code chunk number 51: endyears2

###################################################

jrnls3$endYear2[jrnls3$title == "NHSA Dialog"] <- 2012

###################################################

### code chunk number 52: dataimport-201904-uniquebackupstring201904231822.Rnw:870-871

###################################################

jrnls3$endYear2 <- jrnls3$endYear2 + 1

###################################################

### code chunk number 53: duration1

###################################################

jrnls3$duration2 <- ifelse(jrnls3$statusf == "Active", max(jrnls3$endYear2, na.rm=TRUE) - jrnls3$startYear2 + 1,

ifelse(jrnls3$statusf == "Ceased", jrnls3$endYear2 - jrnls3$startYear2,

NA))

table(jrnls3$duration2, exclude = NULL)

###################################################

### code chunk number 54: duration2

###################################################

getOption("SweaveHooks")[["fig"]]()

barplot(table(jrnls3$duration2), xlab = "Journal lifespan",

ylab = "Count", ylim = c(0, 300))

###################################################

### code chunk number 55: dataimport-201904-uniquebackupstring201904231822.Rnw:898-903

###################################################

j1 <- dim(jrnls3)

jrnls3.checkpoint <- jrnls3

## weird R defect. > 1990 removes NAs, but == 1990 keeps NAs and 1990

jrnls3 <- jrnls3[is.na(jrnls3$startYear2) | jrnls3$startYear2 > 1900, ]

j2 <- dim(jrnls3)

###################################################

### code chunk number 56: older10

###################################################

j1 <- dim(jrnls3)

jrnls4 <- jrnls3[is.na(jrnls3$endYear2) | jrnls3$endYear2 >= 1980, ]

j2 <- dim(jrnls4)

###################################################

### code chunk number 57: statusf2

###################################################

## Create a dummy for failure status. Needed by survival models

jrnls4$failvar <- ifelse(jrnls4$statusf == "Ceased", 1, 0)

###################################################

### code chunk number 58: statusf40

###################################################

j99 <- table(status = jrnls4$status, "failure variable" = jrnls4$failvar, exclude = NULL)

print(xtable(j99), floating=FALSE)

###################################################

### code chunk number 59: cohort1

###################################################

jrnls4$cohort <- cut(as.numeric(jrnls4$startYear2),

breaks = c(1800, 1970, 1980, 1990, 2000, 2010, 2020),

labels = c("bf1970", "1970s", "1980s", "1990s", "2000s", "2010s"),

right = FALSE)

###################################################

### code chunk number 60: cohort2

###################################################

t2 <- addmargins(table("status" = jrnls4$statusf, "cohort" = jrnls4$cohort, exclude = NULL))

t2xt <- xtable(t2)

print(t2xt, include.rownames=FALSE, floating=FALSE)

###################################################

### code chunk number 61: status100

###################################################

## Lets don't show this for now.

t2 <- with(jrnls4[jrnls4$status %in% c("Active", "Ceased"), ], table(statusf, endYear2, exclude = NULL))

t2len <- floor(0.5 * length(t2))

t2a <- t2[ , (t2len-8): t2len]

t2axt <- xtable(t2a)

# t2axt <- xtable(t2a)

print(t2axt, floating=FALSE)

###################################################

### code chunk number 62: savedf

###################################################

## Lower case the variable names, was done in the stata code too

colnames(jrnls4) <- tolower(colnames(jrnls4))

## columns we are sure we never need:

colstodrop <- c("row.names", "coden", "otherfeatures", "price", "toc",

"historictitle", "availableonline", "formatscsv", "languagescsv",

"rss", "from.x", "from.y", "jrnls")

jrnls4[ , colstodrop] <- NULL

fn <- file.path(wdir, paste0("jrnls4"))

fn.csv <- paste0(fn, ".csv")

fn.rds <- paste0(fn, ".rds")

file.backup(fn.csv)

file.backup(fn.rds)

write.csv2(jrnls4, fn.csv, row.names = FALSE)

saveRDS(jrnls4, fn.rds)

###################################################

### code chunk number 63: sessioninfo

###################################################

zz <- "sessionInfo.Rout"

capture.output(sessionInfo(), file = zz, append = FALSE)

if (!is.null(warnings())){

capture.output(warnings(), file = zz, append = TRUE)

}

###################################################

### code chunk number 64: RoptionsRestore

###################################################

## Don't delete this. It puts the interactive session options

## back the way they were. If this is compiled within a session

## it is vital to do this.

options(opts.orig)

par(par.orig)

**dataimport-201904.rnw**

%% LyX 2.2.3 created this file. For more info, see http://www.lyx.org/.

%% Do not edit unless you really know what you are doing.

\documentclass[11pt,letterpaper,english]{extarticle}

\usepackage{lmodern}

\renewcommand{\sfdefault}{lmss}

\renewcommand{\ttdefault}{lmtt}

\usepackage[T1]{fontenc}

\usepackage[utf8]{inputenc}

\setlength{\parskip}{\medskipamount}

\setlength{\parindent}{0pt}

%% no cites here

%% \usepackage[authoryear]{natbib}

\makeatletter

%%%%%%%%%%%%%%%%%%%%%%%%%%%%%% LyX specific LaTeX commands.

\special{papersize=\the\paperwidth,\the\paperheight}

%%%%%%%%%%%%%%%%%%%%%%%%%%%%%% Textclass specific LaTeX commands.

<<echo=F>>=

if(exists(".orig.enc")) options(encoding = .orig.enc)

@

\providecommand*{\code}[1]{\texttt{#1}}

\@ifundefined{date}{}{\date{}}

%%%%%%%%%%%%%%%%%%%%%%%%%%%%%% User specified LaTeX commands.

% Don't remove next commented line, its needed to fool Sweave

%\usepackage{Sweave}

\usepackage[includehead,includefoot,

lmargin=1in,

rmargin=1in,

tmargin=0.75in,

bmargin=1.0in,

headheight=0pt,

headsep=0pt,

marginparwidth=0pt,

footskip=1.5\baselineskip,

]{geometry}

\usepackage{dcolumn}

\usepackage{booktabs}

\input{theme/reportPreambleHeader.tex}

\input{theme/preambleFooter.tex}

\input{theme/reportPreambleSweavel.tex}

\makeatother

\usepackage{babel}

\begin{document}

%% Fill in values of the arguments here,

%% If blanks are needed, must insert value " ~ "

%% If comma needed inside value, wrap in {}.

%% Delete secondauthor and thirdauthor if not needed

\reportsetup{%

author={

lastname=Johnson,

firstname=Paul,

affiliation=CRMDA,

email=pauljohn@ku.edu},

title={Technical Report: Importing the Ulrich's Web Data},

rightlogo={theme/logo.pdf},

}

\reporthdr

% tmpout directory must exist first

<<tmpout, echo=FALSE, include=FALSE, results=hide>>=

if(!dir.exists("tmpout")) dir.create("tmpout", showWarnings=FALSE)

@

% Please leave this code: In document Latex options:

\fvset{listparameters={\setlength{\topsep}{0em}}}

\SweaveOpts{prefix.string=tmpout/t, split=TRUE, ae=FALSE, height=5, width=6}

% Please leave this chunk

<<excludemeRoptions, echo=F, include=F, results=hide>>=

if(!dir.exists("tmpout"))dir.create("tmpout", recursive = TRUE)

opts.orig <- options()

options(device = pdf)

options(width=70, prompt=" ", continue=" ")

options(useFancyQuotes = FALSE)

set.seed(12345)

par.orig <- par(no.readonly=TRUE)

pjmar <- c(5.1, 5.1, 1.5, 2.1)

options(SweaveHooks=list(fig=function() par(mar=pjmar, ps=10)))

pdf.options(onefile=FALSE, family="Times", pointsize=10)

@

\begin{abstract}

\noindent

This is a report on procedures for data extraction and

re-classifcation in the project entitled, ``Did the NIH public

access policy really kill biomedical journals?'' by A. Townsend

Peterson, Paul E. Johnson, Narayani Barve, Ada Emmett, Marc

Greenerg, Josh Bolick and Huijie Qiao. It describes the procedures

used to download, filter and classify journals by subject.

A companion R file that has the same name as this report, with the

suffix ``.R'', is available.

\end{abstract}

<<setup, echo=F, results=hide>>=

ddir <- "../../data"

wdir <- "../../workingdata"

odir <- "output"

if(!file.exists(odir)) dir.create(odir)

fn1 <- "all_ulrich_data-20190415.rdata"

fn2 <- "filtered-20190416.rdata"

#fn2 <- "filtered_all_journals_20190417.RData"

today <- format(Sys.time(), "%Y%m%d")

fn1.tex <- "all\\\\_ulrich\\\\_data-20190415.rdata"

fn2.tex <- "filtered-20190416.rdata"

##fn2.tex <- "filtered\\\\_all\\\\_journals\\\\_20190417.RData"

@

\section{Data Extraction}

The records for publications were extracted by structured query

language (SQL) requests on the \emph{Ulrich's Web} applications programming

interface (API).

The download process results in two files, ``\Sexpr{fn1.tex}'' and\\

``\Sexpr{fn2.tex}''. The former is a comprehensive account of the

records available from \emph{Ulrich's Web}, which offers many serial

publications, some of which have ceased publication long in the past.

The variables in which we are most interested are the start year and

the subject classification. The latter file includes the information

about journals that have ceased publication. It has the year in which

the journal was discontinued.

\section{Data Import}

<<alldata, echo = F, results = hide>>=

##jrnls.0 <- readRDS(file.path(ddir, fn1))

load(file.path(ddir, fn1))

jrnls.0 <- df

j0 <- dim(jrnls.0)

@

<<endyeardata1, echo = F, results = hide>>=

# jrnls_endyear <- readRDS(file.path(ddir, fn2))

load(file.path(ddir, fn2))

#jrnls_endyear <- df_filter_all_journal

jrnls_endyear <- df_filter

j1 <- dim(jrnls_endyear)

## key identifiers, called matchvars.

matchvars <- c("titleId", "title", "startYear", "status", "formats")

## Keep only the variables we need from jrnls_endyear

keepers <- c(matchvars, "endYear")

jrnls_endyear <- jrnls_endyear[ , keepers]

@

There were \Sexpr{j0[1]} rows of information in ``\Sexpr{fn1.tex}''.\\

In ``\Sexpr{fn2.tex}'' there are \Sexpr{j1[1]} rows.

\section{Preliminary Row Filtering}

<<echo=F>>=

## Did not make latex work here, though

library(xtable)

@

<<jrnlsrowfilter1, echo=F>>=

jrnls_dupes <- duplicated(jrnls.0[ , matchvars])

jrnls.0 <- jrnls.0[!jrnls_dupes, ]

j2 <- dim(jrnls.0)

@

There are some rows in each file that are redundant. A record is

considered redundant if the following items are identical: titleId,

title, startYear, status, formats. After removing duplicates, the row

count drops from \Sexpr{j0[1]} to \Sexpr{j2[1]}.

<<endyeardata2, echo=F, results=hide>>=

## There are no duplicated rows on the match vars:

jrnls_endyear_dupes <- duplicated(jrnls_endyear[ , matchvars])

table(jrnls_endyear_dupes, exclude = NULL)

@

There are duplicated rows that remain because many journals are

disseminated in more than one format. One example is

\emph{Particle Accelerators}, which appears in four rows in both of

the data sets.

<<endyeardata3, echo=F, results=tex>>=

j99 <- jrnls_endyear[jrnls_endyear$title == "Particle Accelerators", ]

print(xtable(j99), floating=FALSE)

@

De-duplicating these entries is discussed below in section \ref{sec:dedupe}

\section{Merge}

<<merge1, echo=F, include=F>>=

## Add markers to facilitate diagnostics on unmatched cases

jrnls.0$from.x = TRUE

jrnls_endyear$from.y <- TRUE

jrnls <- merge(jrnls.0, jrnls_endyear,

by = matchvars,

sort = FALSE, all.x = TRUE, all.y = TRUE)

@

<<merge1a, echo=F,results=hide>>=

j3 <- dim(jrnls)

@

Next we integrate the information for the journals that have been

closed. This is a horizontal ``join'' operation. As a merge

diagnostic, we find that all of the titles that appear in the second

data set are matched in the first one. In other words, the merge is a

success.

<<merge2, echo=F, results=hide>>=

## merge diagnostic:

t1 <- table("from Ulrich" = jrnls$from.x, "from endYear data" = jrnls$from.y, exclude = NULL)

t1xt <- xtable(t1)

print(t1xt, floating=FALSE)

@

% If the journals in ``\Sexpr{fn2.tex}'' and ``\Sexpr{fn1.tex}'', are aligned

% perfectly, this table will have only one row. One source of concern is

% that many of the rows in which the status of the journal is ``Ceased''

% do not include information about the endYear variable. We will return

% to this subject in section \ref{sec:startend}.

\section{Filter Rows}

\subsection{Eliminate by publication type}

We further limit our attention to publication content types that

are classified by \emph{Ulrich's} as ``Academic / Scholarly'', have

serialTypes equal to ``Journal'', and are published in the United States.

<<jrnlsrowfilter2, echo=F, results=hide>>=

# Restrict to US, Academic/Scholarly journals

jrnls <- jrnls[jrnls$contentTypes == "Academic / Scholarly", ]

j4 <- dim(jrnls)

jrnls <- jrnls[jrnls$country == "United States", ]

j5 <- dim(jrnls)

jrnls <- jrnls[jrnls$serialTypes == "Journal", ]

j6 <- dim(jrnls)

@

We began this phase with \Sexpr{j3[1]} rows of journal information. Limiting

our attention to the content type ``Academic / Scholarly'' reduced that

to \Sexpr{j4[1]}. The requirement that the country be ``United States''

reduced the number of journal rows to \Sexpr{j5[1]}. And by excluding any

serial types that are not ``Journal'', we arrive at \Sexpr{j6[1]} rows.

\subsection{Duplicate Titles}

\label{sec:dedupe}

<<dupecheck1, echo=F, results=hide>>=

dupecheck <- duplicated(jrnls[ , c("title")])

table(dupecheck, exclude = NULL)

titledupes <- jrnls[dupecheck, "title"]

jrnls_dupes <- jrnls[jrnls$title %in% titledupes, ]

jrnls_dupes <- jrnls_dupes[order(jrnls_dupes$title, jrnls_dupes$formats), ]

(nofdupes <- length(unique(jrnls_dupes$title)))

## The first 50 repeated title lines

head(jrnls_dupes$title, 50)

## see them interactively

##View(head(jrnls_dupes, 200))

uniquetitles <- length(unique(jrnls$title))

@

Now we confront the problem that some titles in the collection are

represented by several rows. This occurs because a journal may begin

with a print version, which is later accompanied by (or replaced by)

an online or microfilm version. In our analysis, we do not want to

treat all of these as separate journals. The total number of unique

values for journal title is \Sexpr{uniquetitles} and among them there

are \Sexpr{nofdupes} journal titles with more than one row of

information.

As a litmus test, we will monitor the journal \textit{Zygon}. From the

outset, we have:

<<zygonexample, echo=F, results=tex>>=

jrnlbytitle <- split(jrnls, f = list(jrnls$title))

j99 <- jrnlbytitle[["Zygon"]][ , c("titleId", "title", "startYear", "formats", "endYear")]

print(xtable(j99), floating=FALSE)

@

The corrected data for \emph{Zygon} should have

one row, with \code{startYear} 1966 and \code{endYear} NA.

The data reduction method is to split the data by row blocks, one for

each observed title. Then sift through the rows to find out if there

is information in them that is unique. We created a function called

\code{delteuninformativerows} that can look at the repeated lines and

eliminate the ones that contribute no information. This is available

in the R code companion file.

<<dupefix1, echo=F, results=hide>>=

## This function will receive a block and then delete

## repeated rows, using various kinds of guessing

## that are explained within

deleteuninformativerows <- function(X){

if (NROW(X) == 1) return(X)

## Find rows where both startYear and endYear are uninformative, and

## delete those rows as long at it leaves at least 1 good row

X$removenow <- ifelse(X$startYear %in% c("", NA) & X$endYear %in% c("", NA), TRUE, FALSE)

if (NROW(X) - sum(X$removenow) >= 1){

X <- X[!X$removenow, , drop = FALSE]

} else {

## It wants to delete all of the rows, but only delete N - 1

ntoremove <- sum(X$removenow)

## remove rows 1 to ntoremove - 1

X <- X[-seq(1, ntoremove -1), , drop = FALSE]

}

if (NROW(X) == 1) {

X[ , "removenow"] <- NULL

return(X)

}

## That killed off the empty "online" and "microform" rows.

##

## Now get rid of rows that have startYear like "19??";

## If has question mark or "", startYearn will become NA

X$startYearn <- as.numeric(X$startYear)

X$removenow <- ifelse(X$startYearn %in% c("", NA) & X$endYear %in% c("", NA), TRUE, FALSE)

## If removal does not create a 0 row journal do it

if (NROW(X) - sum(X$removenow) >= 1){

X <- X[!X$removenow, , drop = FALSE]

} else {

## It wants to delete all of the rows, but only delete N - 1

ntoremove <- sum(X$removenow)

## remove rows 1 to ntoremove - 1

X <- X[-seq(1, ntoremove -1), , drop = FALSE]

}

if (NROW(X) == 1){

X[ , c("startYearn", "removenow")] <- NULL

return(X)

}

## Still have multiple rows, so keep only one with smallest

## startYear. Only do this if endYear is uninformative

## If startYear is not all NA

if(any(!is.na(X$startYearn))){

startLowest <- which.min(X$startYearn)

if (length(startLowest) == 1 && length(unique(X$endYear) < 2)){

X <- X[startLowest, , drop = FALSE]

}

if (NROW(X) == 1){

X[ , c("startYearn", "removenow")] <- NULL

return(X)

}

}

## Some cases have 2 rows with contradictory startYear information

## in a format like c("19??", "") or c("198?", "19??").

## This arises in 6 journal

## cases, in all of which startYear is "" or has a "?". If

## there are different values, we don't care much because

## unknown startYear will cause exclusion later. So just keep

## one row.

if(length(unique(X$startYear)) > 1 & all(is.na(as.numeric(X$startYear)))){

X <- X[1, ]

}

## Previous eliminates all but 2 sets of repeats. These

## journals have identical startYear == 19?? and given endYear.

## if rows have same info, keep first one.

if(length(unique(X$startYear)) == 1 & length(unique(X$endYear)) == 1){

X <- X[1, ]

}

X[ , c("startYearn", "removenow")] <- NULL

X

}

@

<<dupefix2, echo=F, results=hide>>=

xxx <- lapply(jrnlbytitle, deleteuninformativerows)

## Warnings are harmless, they concern "19??" turning to NA by

## as.numeric()

jrnls2 <- do.call("rbind", xxx)

## The row names become a distraction

rownames(jrnls2) <- NULL

j7 <- dim(jrnls2)

@

<<dupecheck2, echo=F, results=hide>>=

## This process required several cycles to clean

## the logic in the function above.

dupecheck <- duplicated(jrnls2[ , c("title")])

titledupes <- jrnls2[dupecheck, "title"]

if(length(titledupes) > 0){

jrnls2_dupes <- jrnls2[jrnls2$title %in% titledupes, ]

jrnls2_dupes <- jrnls2_dupes[order(jrnls2_dupes$title, jrnls2_dupes$formats), ]

## If jrnls_dupes has any rows, then we need to go check

print("FAILURE: There are repeated journal titles:")

jrnls2_dupes$title

}

@

Using a filtering algorithm, we reduce the number of data rows to

\Sexpr{j7[1]}. The de-duplication doublecheck indicates we were successful, there are

no more titles with more than one row. For example, the result for

\textit{Zygon} is

<<zygon2, echo=F,results=tex>>=

t10 <- jrnls2[jrnls2$title == "Zygon", c("title", "startYear", "formats", "endYear")]

t10xt <- xtable(t10)

print(t10xt, floating=FALSE)

@

\subsection{Unsolved duplicate title problems}

\label{sec:unsolved}

In the previous effort, we concentrated on eliminating repeated

titles. That meant titles that were exactly the same. We still have a

second kind of duplication. There are journals with multiple rows

that should be compressed into 1 row, such as:

\begin{enumerate}

\item The Journal of Musculoskeletal Medicine (Online)

\item The Journal of Musculoskeletal Medicine (Print)

\end{enumerate}

or

\begin{enumerate}

\item The Journal of Contemporary Health Law and Policy (Online)

\item The Journal of Contemporary Health Law and Policy (Print)

\end{enumerate}

There are additional complications where the startYears for the two

versions of the journals differ. Clearly, we want to keep the first

startYear value, and the latest endYear. One (among many) interesting

examples is the journal \emph{Northeastern Naturalist}:

<<mordupe1, echo=F, results=tex>>=

j99 <- jrnls2[grep("Northeastern Naturalist" , jrnls2$title), c("title", "status", "startYear", "endYear")]

print(xtable(j99), floating=FALSE)

@

There are two obvious problems. First, there are two

rows where we want one. Second, the start year includes a question

mark. If any of the records for a journal has a valid year

for the start or end year, we will use that information.

%An inspection of the

% journal's \href{https://www.eaglehill.us/NENAonline/NENAregular.shtml}{Website}

% shows that in 2018 they published volume 25 and it appears they offer

% yearly volumes, so the start year for the journal might be 1993 to be

% $2018 - 25$.

\emph{Northeastern Naturalist} (NN) started in 1993 as a print

journal, but after a brief while, it became an online online. As a

result, we combine the rows and treat the starting year as 1993.

<<mordupe3, echo=F, results=hide>>=

## Shadi's suggestion

## jrnls2$title.chopped <- trimws(gsub("\\(.*\\)", "", jrnls2$title))

## dupedtitles <- jrnls2$title.chopped[duplicated(jrnls2$title.chopped)]

jrnls2$title.chopped <- trimws(gsub("[:(:]Print[:):]|[:(:]Online[:):]", "", jrnls2$title))

dupedtitles <- jrnls2$title.chopped[duplicated(jrnls2[ , c("title.chopped", "publisher", "subject")])]

dupecount <- length(unique(dupedtitles))

jrnls2$titleisdupe <- jrnls2$title.chopped %in% dupedtitles

table(jrnls2$titleisdupe)

@

There are \Sexpr{dupecount} journals that have titles that are

duplicated, except for the inclusion of the word ``(Print)'' and

``(Online)'' in the titles. We filter those by requiring the publisher

and the subject of the journals must be identical.

<<echo=F,results=hide>>=

## Make inspection easier by sorting

jrnls2 <- jrnls2[order(jrnls2$title.chopped), ]

@

<<mordupe4, echo=F, results=hide>>=

jrnls2.split <- split(jrnls2, f = jrnls2$title.chopped)

@

<<mordupe5, echo=F, results=hide>>=

xxx <- lapply(jrnls2.split, deleteuninformativerows)

## Warnings are harmless, they concern "19??" turning to NA by

## as.numeric()

jrnls3 <- do.call("rbind", xxx)

j8 <- dim(jrnls3)

@

Using a de-duplication strategy (which can be inspected in the

accompanying R file), we have reduced the number of rows to

\Sexpr{j8[1]}. The preliminary spot check on three of the

journals mentioned above is encouraging:

<<mordupe6, echo=F, results=tex>>=

k1 <- jrnls3[grep("Northeastern Naturalist" , jrnls3$title), c("status", "startYear", "endYear")]

k2 <- jrnls3[grep("The Journal of Contemporary Health Law and Policy" , jrnls3$title), c("status", "startYear", "endYear")]

k3 <- jrnls3[grep("The Journal of Musculoskeletal Medicine" , jrnls3$title), c("status", "startYear", "endYear")]

kk <- rbind(k1, k2, k3)

print(xtable(kk), floating=FALSE)

@

% Here is one way to check:

<<cleanup10, echo=F>>=

jrnls3[ , c("title.chopped", "titleisdupe")] <- NULL

jrnls3[ , c("from.x", "from.y")] <- NULL

@

<<mordupe10, eval=F, echo=F, results=hide>>=

## How I conducted visual inspection of titles that seem to be

## same, except for parentheses. This shows very few duplicated

## journals remain

jrnls3t <- jrnls3

jrnls3t$title.chopped <- trimws(gsub("\\(.*\\)", "", jrnls3t$title))

dupedtitles <- jrnls3t$title.chopped[duplicated(jrnls3t$title.chopped)]

(dupecount <- length(unique(dupedtitles)))

jrnls3t$titleisdupe <- jrnls3t$title.chopped %in% dupedtitles

if(interactive()) View(jrnls3t[jrnls3t$titleisdupe , c("title", "title.chopped", "publisher")])

rm(jrnls3t)

@

\section{Assign Subject Classifications}

The \emph{Ulrich's Web} system uses a multiple keword classification system. In

this section, we discuss our procedure for sorting through the

classification provided by \emph{Ulrich's Web}. We create both a ``primary''

subject classification for each journal, as well as indicators for

each journal indicating if it might belong to the families defined by

agriculture, bio-medical research, engineering, social science,

natural science, or physical science.

\subsection{Subject data format}

\label{sec:subjectformat}

<<ajms, echo=F, results=hide>>=

ajms.subj <- jrnls2[jrnls2$title == "American Journal of Materials Science", "subject"]

@

The subject listings in the \emph{Ulrich's Web} data base are multiple-topic

indicators separated by punctuation, commas, colons, and dashes. We

have exerted great care to parse the punctuation correctly.

Consider the \emph{American Journal of Materials Science}

(\emph{AJMS}), for which the subject string is

<<ajmssubj2, echo=F>>=

cat(ajms.subj)

@

This case is useful as an illustration of the complicated format

of the subject information. The subject string uses

commas both as a separator for large items and also for punctuation

within the items. The commas that are not followed by white space are terminators for

subjects. The AJMS subject heading should be seen as

\begin{enumerate}

\item CERAMICS, GLASS AND POTTERY

\item ENGINEERING - CHEMICAL ENGINEERING

\item ENGINEERING - ENGINEERING MECHANICS AND MATERIALS

\item METALLURGY

\end{enumerate}

<<jope1, echo=F, results=hide>>=

jope.subj <- jrnls2[grep("^Journal of Political Economy", jrnls2$title), "subject"]

@

Another interesting case is the \emph{Journal of Political Economy} (\emph{JOPE}), for

which the subject string is

<<jope2, echo=F>>=

cat(jope.subj)

@

That subject string should be understood as

\begin{enumerate}

\item BUSINESS AND ECONOMICS

\item POLITICAL SCIENCE

\end{enumerate}

<<trackers, echo=F, results=hide>>=

trackme <- c("Journal of Political Economy", "American Journal of Materials Science")

trackmeid <- c("737879", "48302")

@

The primary subject identifiers are categorized into larger sets by

a matching system using a taxonomy that was designed by our subject

matter experts.

\def\Rsize{\scriptsize}

<<subgen, echo=F>>=

subtypes <-

list(BIOMED = c('MEDICAL SCIENCES', 'PHARMACY AND PHARMACOLOGY',

'PSYCHOLOGY', 'PUBLIC HEALTH AND SAFETY',

'HEALTH FACILITIES AND ADMINISTRATION'),

NATSCI = c('BIOLOGY', 'GEOGRAPHY',

'EARTH SCIENCES', 'ENVIRONMENTAL STUDIES',

'FISH AND FISHERIES', 'FORESTS AND FORESTRY',

'PALEONTOLOGY', 'CONSERVATION'),

PHYSCI = c('CHEMISTRY', 'ASTRONOMY', 'MATHEMATICS', 'PHYSICS',

'STATISTICS', 'METEORLOGY'),

ENGTECH = c('AERONAUTICS AND SPACE FLIGHT', 'COMPUTERS',

'ENGINEERING', 'ENERGY', 'TECHNOLOGY',

'LIBRARY AND INFORMATION SCIENCES'),

SOCSCI = c('ANTHROPOLOGY', 'SOCIAL SCIENCES', 'SOCIOLOGY',

'ARCHAEOLOGY', 'POLITICAL SCIENCE',

'POPULATION STUDIES', 'SOCIAL SERVICES AND WELFARE'),

AGRICUL = c('AGRICULTURE'))

@

<<subgenbeauty, echo=F, results=tex>>=

keywords = sapply(subtypes, paste, collapse = ", ")

fakedf <- data.frame("subject.areas" = names(keywords),

keywords = keywords)

fakedfxt <- xtable(fakedf, align = c("l", "l", "p{5in}"))

print(fakedfxt, include.rownames=F, floating=FALSE)

@

\subsection{Our Classification Strategy}

\label{sec:subjectalt}

We check each of the separate elements in the \emph{Ulrich's} subject

data. In the case of the \emph{AJMS}, the original subject

classification is reported as:

<<echo=F>>=

jrnls3[jrnls3$title %in% trackme , c("subject")]

@

First, we parse the subject string at the comma that is not followed

by a space, so that the subject for the AJMS is seen as 4

elements.

<<newsep1, echo=F,results=hide>>=

## Insert a SEParator where we think it should be

subjnew1 <- gsub("(,(?=\\S))", "__SEP__", jrnls3$subject, perl = TRUE)

subjnewsplit <- strsplit(subjnew1, split = "__SEP__", fixed = TRUE)

## Use titleId as names

names(subjnewsplit) <- jrnls3$titleId

@

The detailed specifier after the long dash is then stripped. The

remaining elements are matched against our subjects.

<<newsep2, echo=F>>=

subjnewsplit[["737879"]]

@

% We delete the material that the dash (`` - ``) and colon (`` : ``) and

% following information to arrive at the candidates for matching with

% the topic dictionary. \textbf{ATTENTION: we need Town to confirm this idea}

<<newsep3, echo=F>>=

subjnewsplit2 <- lapply(subjnewsplit, function(x) trimws(gsub("[:|-].*", "", x)))

names(subjnewsplit2) <- names(subjnewsplit)

subjnewsplit2[["737879"]]

@

We classify journals from ``left to right.'' If there is a match in

the first term, we create a classification. When the first element in

the subject is not matched, then the subsequent elements can be

considered.

<<newsep4, echo=F>>=

matches <- matrix(NA, ncol = length(subtypes), nrow = length(subjnewsplit2),

dimnames = list(names(subjnewsplit2), names(subtypes)))

for(i in names(subtypes)){

#browser()

withbars <- paste0(subtypes[[i]], collapse = "|")

reslt <- vapply(subjnewsplit2, function(x){

itemmatch <- grep(withbars, x, perl=TRUE, ignore.case=TRUE)

itemmatch <- if(length(itemmatch) == 0) NA else min(itemmatch)

}, integer(1)

)

matches[ ,i] <- reslt

}

@

We create a matrix in which the integer values represent which

terms are matched. Consider a few rows:

<<newsep5, echo=F, results=tex>>=

m1 <- matches[c("164399", "800201", "592789", trackmeid), ]

m1xt <- xtable(m1)

print(m1xt, floating=FALSE)

@

The rows represent journals, the columns represent classification. If

there is a 1 in a cell, it means that the first term in the subject

vector was matched by a keyword within our classification table. A 2

indicates that the second word in the \emph{Ulrich's} classification matched

one of our topics.

Following this procedure, we end up with individual indicator

variables, one for each subject area (coded 0 or 1), as well as a

``primary'' subject indicator, which is the matching category which

has the lowest score in the match matrix. If the whole row is

missing--filled with NA values--then no subject grouping is assigned.

The \emph{American Journal of Materials Science} is the second-to-last

line in the matrix displayed above. There is no first term match, but

the second term is matched by an engineering term. Hence, we classify

AMJS as an ENGTECH journal.

The \emph{Journal of Political Economy} is the last line in the

matrix. Note that it has no first term matches, but the second subject

topic term is matched by a social science keyword. We classify this as

a SOCSCI journal.

<<newsep6, echo=F>>=

jrnls3$subject.grouped2 <- apply(matches, 1, function(x){

if(all(is.na(x))) NA else colnames(matches)[which.min(x)]

})

jrnls3$subject.grouped2 <- toupper(jrnls3$subject.grouped2)

@

Using this method, the number of journals that falls into the

designated catetories is as follows:

<<newsep8, echo=F, results=tex>>=

t5 <- addmargins(table("Count" = jrnls3$subject.grouped2, exclude = NULL))

# t5[-c(7,8), 7]

t5xt <- xtable(t5, digits=0)

print(t5xt, floating = FALSE)

@

The Sum is greater than the sample size because some

journals are classified in more than one subject area.

<<echo=F, results=hide>>=

rownames(jrnls3) <- jrnls3$titleId

matches[is.na(matches)] <- 0

matches[matches > 1] <- 1

jrnls3 <- merge(jrnls3, matches, by = "row.names", all=TRUE)

@

<<newsep9, echo=F, results=hide>>=

## group classification snapshot file

keepers <- c("titleId", "title", "subject", "AGRICUL", "BIOMED", "ENGTECH", "NATSCI", "PHYSCI", "SOCSCI")

ofn <- paste0("jrnls-subjects-", today, ".csv")

library(kutils)

file.backup(file.path(odir, ofn))

write.csv(jrnls3[ , keepers], file = file.path(odir, ofn), row.names = FALSE)

@

% To facilitate inspection, we created a CSV file \Sexpr{ofn} that

% has these columns:

% <<echo=F>>=

% cat(keepers)

% @

\section{Creating Indicators to Facilitate Analysis}

\subsection{A marker for `refereed' and `reviewed' journals}

<<refereedfilter, echo=F, results=hide>>=

jrnls3$rrev <- factor(ifelse(jrnls3$refereed == "TRUE" & jrnls3$reviewed == "TRUE", "both",

ifelse(jrnls3$refereed == "TRUE", "refereed",

ifelse(jrnls3$reviewed == "TRUE", "reviewed",

"neither"))), levels = c("refereed", "reviewed", "both", "neither"))

@

The data has two classifier variables, ``refereed'' and ``reviewed'',

which are distilled into one indicator in our data collection. The

tabulation is as follows, for each subject grouping.

<<refereedfilter2, echo=F, results=tex>>=

t3 <- table("refeered" = jrnls3$rrev, "single-subject grouping" = jrnls3$subject.grouped2, exclude = NULL)

t3xt <- xtable(t3)

print(xtable(t3xt), floating=FALSE)

@

If we exclude the journals that are neither refereed nor reviewed, we

reduce the number of subject-classified journals by

\Sexpr{sum(t3[4,-7])} journals.

\section{A marker for status: Active or Ceased}

\label{sec:active}

The journal's status can be scored with a number of labels.

Almost all journals are Active or Ceased, as we see in the following:.

<<echo=F, results=tex>>=

t1 <- table("Count" = jrnls3$status)

print(xtable(t1), floating = FALSE)

@

We exclude from consideration the journals that have status ``Announced

Never Published'', ``Forthcoming'', and so forth. We create an indicator

variable \code{statusf} which is missing for all status values except

Active and Ceased.

<<echo=F, results=tex>>=

jrnls3$statusf <- factor(jrnls3$status, levels = c("Active", "Ceased"))

t1 <- table("status original" = jrnls3$status, "status new version" = jrnls3$statusf, exclude = NULL)

print(xtable(t1), floating = FALSE)

@

\section{Start and End year for journals}

\label{sec:startend}

In order to conduct a survival analysis, we need the start years for

all journals under consideration. We also need the termination year

for all of the Ceased journals. In the startYear summary, the first

value is blank and there are several years including ``?''. Those

cases are omitted from the analysis, but for record keeping we retain

both the original information about the start and end year as well

as the cleaned version. We end up with 2 sets of year variables,

<<createyearvars, echo=F, results=hide>>=

## Years with non-nonnumeric values are set as missings

jrnls3$startYear2 <- as.integer(jrnls3$startYear)

jrnls3$endYear2 <- as.integer(jrnls3$endYear)

@

\begin{enumerate}

\item startYear: original data, includes blanks and ``?'' values from input data

\item startYear2: all non-integer values converted to missing values

\item endYear: original data, includes blanks and ``?'' values from input data

\item endYear2: endYear + 1, all non-integer values converted to missing values

\end{enumerate}

\label{sec:endyears}

In the data for journal termination date, there is an obvious

typographical error indicating that ``NHSA Dialog'' was in 3012.

That value should be 2012.

<<endyears2, echo=F>>=

jrnls3$endYear2[jrnls3$title == "NHSA Dialog"] <- 2012

@

There are some journals for which the \code{startYear} and

\code{endYear} are identical. That happens because the variable

\code{endYear} is the last year of publication, rather then the year

after termination. Hence, for calculating a journal's age (or

duration of survival), we use $endYear + 1$. %'

<<echo=F, echo=F, results=hide>>=

jrnls3$endYear2 <- jrnls3$endYear2 + 1

@

The variable ``duration'' will represent the number of years that a

journal existed before closure. For journals that have not closed, the

``duration'' is the difference between the maximum value of the

observed end year plus 1 and the journal's start year.

<<duration1, echo=F, results=hide>>=

jrnls3$duration2 <- ifelse(jrnls3$statusf == "Active", max(jrnls3$endYear2, na.rm=TRUE) - jrnls3$startYear2 + 1,

ifelse(jrnls3$statusf == "Ceased", jrnls3$endYear2 - jrnls3$startYear2,

NA))

table(jrnls3$duration2, exclude = NULL)

@

<<duration2, echo=F, fig=T>>=

barplot(table(jrnls3$duration2), xlab = "Journal lifespan",

ylab = "Count", ylim = c(0, 300))

@

\subsection{Exclude Very Old Journals}

\label{sec:excludeold}

<<echo=F>>=

j1 <- dim(jrnls3)

jrnls3.checkpoint <- jrnls3

## weird R defect. > 1990 removes NAs, but == 1990 keeps NAs and 1990

jrnls3 <- jrnls3[is.na(jrnls3$startYear2) | jrnls3$startYear2 > 1900, ]

j2 <- dim(jrnls3)

@

The oldest journal in our collection began in 1758. We have limit our

consideration to journals that were created before 1900. Doing

so removes from consideration \Sexpr{j1[1] - j2[1]} journals.

\subsection{Drop journals that closed before 1980}

\label{sec:dropold}

<<older10, echo=F, results=hide>>=

j1 <- dim(jrnls3)

jrnls4 <- jrnls3[is.na(jrnls3$endYear2) | jrnls3$endYear2 >= 1980, ]

j2 <- dim(jrnls4)

@

There are a handful of journals that have end years before 1980. We

exclude them from further consideration. The omission of journals that

closed before 1980 affected \Sexpr{j1[1] - j2[1]} rows of information.

\subsection{Variables for Duration Analysis: failvar, cohort}

For survival analysis, we need a variable indicating whether a journal

has ceased to exist before the end of the period under

consideration. This new variable, \code{failvar}, is coded 0 if no

``event'' (closure) occurred, and 1 if a closure event occurred.

<<statusf2, echo=F, results=hide>>=

## Create a dummy for failure status. Needed by survival models

jrnls4$failvar <- ifelse(jrnls4$statusf == "Ceased", 1, 0)

@

The failure variable is coded as missing for all of the status values

except Active or Ceased.

<<statusf40, echo=F, results=tex>>=

j99 <- table(status = jrnls4$status, "failure variable" = jrnls4$failvar, exclude = NULL)

print(xtable(j99), floating=FALSE)

@

We have experimented with various cohort groupings for the

journals. The primary investigation isolated journals that existed at

or after 1980. As a result, the ones that were created before, say,

1970, are not a representative sample of all journals. The ones

created after 1980, however, are more likely to be representative. As

a result, it seems wise to isolate journals into several date-of-birth

cohorts so that we can track their survival separately.

The variable \code{cohort} is the decade-based tabulation

<<cohort1, echo=F, results=hide>>=

jrnls4$cohort <- cut(as.numeric(jrnls4$startYear2),

breaks = c(1800, 1970, 1980, 1990, 2000, 2010, 2020),

labels = c("bf1970", "1970s", "1980s", "1990s", "2000s", "2010s"),

right = FALSE)

@

One striking fact is apparent from the cross tabulation of the new

cohorts with the status indicator: There has been a prolific expansion

in the number of journals since 2000.

<<cohort2, echo=F, results=tex>>=

t2 <- addmargins(table("status" = jrnls4$statusf, "cohort" = jrnls4$cohort, exclude = NULL))

t2xt <- xtable(t2)

print(t2xt, include.rownames=FALSE, floating=FALSE)

@

<<status100, echo=F, results=hide>>=

## Lets don't show this for now.

t2 <- with(jrnls4[jrnls4$status %in% c("Active", "Ceased"), ], table(statusf, endYear2, exclude = NULL))

t2len <- floor(0.5 * length(t2))

t2a <- t2[ , (t2len-8): t2len]

t2axt <- xtable(t2a)

# t2axt <- xtable(t2a)

print(t2axt, floating=FALSE)

@

One point of concern is that the data on the last year of publiction

for \Sexpr{t2[length(t2)]} of the journals that have ceased publication.

\section{Exported Data snapshots}

\label{sec:save}

The process of filtering and reclassifying that is

described in this report generates working data files in both comma

separated variable (CSV) and R data serialization (RDS) formats

(jrnls4.\{csv,rds\}).

<<savedf, echo=F, results=hide>>=

## Lower case the variable names, was done in the stata code too

colnames(jrnls4) <- tolower(colnames(jrnls4))

## columns we are sure we never need:

colstodrop <- c("row.names", "coden", "otherfeatures", "price", "toc",

"historictitle", "availableonline", "formatscsv", "languagescsv",

"rss", "from.x", "from.y", "jrnls")

jrnls4[ , colstodrop] <- NULL

fn <- file.path(wdir, paste0("jrnls4"))

fn.csv <- paste0(fn, ".csv")

fn.rds <- paste0(fn, ".rds")

file.backup(fn.csv)

file.backup(fn.rds)

write.csv2(jrnls4, fn.csv, row.names = FALSE)

saveRDS(jrnls4, fn.rds)

@

<<sessioninfo, include=F>>=

zz <- "sessionInfo.Rout"

capture.output(sessionInfo(), file = zz, append = FALSE)

if (!is.null(warnings())){

capture.output(warnings(), file = zz, append = TRUE)

}

@

<<RoptionsRestore, echo=F, include=F>>=

## Don't delete this. It puts the interactive session options

## back the way they were. If this is compiled within a session

## it is vital to do this.

options(opts.orig)

par(par.orig)

@

\end{document}

**add_end_year.R**

#-------------------------------------------------------

# Because the Ulrich table has no endYear information,

# we need to download the detail page for each journal,

# and extract the endYear from it.

# This script is used to extract the endYear.

#------------------------------------------------

setwd("~/ulrich20190407")

# load the Ulrich records

load("data/filtered-20190416.rdata")

df<-list.files("data_details")

for (i in c(1:length(df))){

print(i)

#get the detail page of the journal

fileName<-sprintf("data_details/%s", df[i])

#extract titleId from the filename

titleId<-gsub('.html', '', df[i])

#read all characters from the html file

f<-readChar(fileName, file.info(fileName)$size, useBytes=T)

#remove all junk characters

f<-gsub('[\n\r]','',f)

f<-gsub('[ ]','',f)

f<-gsub('[\\\"]','',f)

#extract the endYear data from the html page, and update to the data set.

f1<-strsplit(f, "<spanid=title_endYear>EndYear</span></th><td>")

if (length(f1[[1]])==2){

f2<-strsplit(f1[[1]][2], "</td>")

year<-f2[[1]][1]

df_filter[which(df_filter$titleId==titleId),]$endYear<-year

}

}

#save the results.

save(df_filter, file="data/filtered-20190416.rdata")
