# Supplementary material for: The NIH public access policy did not harm biomedical journals
Source: PLoS Biol. 2019 Oct 23;17(10):e3000352. doi: 10.1371/journal.pbio.3000352 (PMC6808382; doi:10.1371/journal.pbio.3000352)
Supplement: S1 Data — (PDF) [file pbio.3000352.s005.pdf]

Supplementary Table 1. Birth rates for journals, expressed as births per 1000 journals

Year	All journals	AGRICUL	BIOMED	ENGTECH	NATSCI	PHYSCI	SOCSCI
1980	50.26	0.00	60.53	58.82	34.91	56.39	40.98
1981	51.33	0.00	58.22	55.56	55.42	28.57	49.87
1982	35.70	0.00	45.31	35.09	38.81	27.87	17.50
1983	41.74	43.48	55.79	33.90	39.56	33.90	24.57
1984	36.60	41.67	46.03	36.07	23.26	26.23	28.78
1985	31.99	20.00	39.36	28.48	18.67	22.44	30.30
1986	43.64	19.61	55.91	55.38	26.48	21.94	45.25
1987	39.51	57.69	47.05	35.09	31.81	27.61	36.80
1988	43.71	18.18	55.69	39.55	38.61	47.76	29.23
1989	36.88	17.86	45.07	40.87	27.88	22.79	34.55
1990	39.52	35.71	41.01	31.41	34.67	44.57	45.19
1991	38.81	0.00	40.37	45.69	33.51	50.67	28.20
1992	32.87	34.48	27.08	51.09	34.13	33.08	27.47
1993	30.61	33.33	30.51	53.24	31.40	37.04	8.91
1994	34.03	64.52	38.23	46.15	24.04	33.41	24.73
1995	35.94	30.77	37.51	46.41	26.60	39.26	31.09
1996	30.73	14.93	34.97	46.46	27.52	20.00	23.49
1997	30.67	29.41	27.86	40.70	23.85	28.32	36.07
1998	25.75	57.14	24.25	31.72	26.24	16.99	19.05
1999	28.03	13.70	33.86	28.93	26.99	20.88	21.84
2000	28.42	0.00	34.50	26.60	19.42	14.31	29.14
2001	24.47	0.00	23.37	31.30	17.76	22.27	23.92
2002	27.74	28.57	24.57	33.96	24.32	21.87	33.77
2003	24.22	0.00	24.64	28.15	21.11	21.40	17.17
2004	25.24	13.89	16.19	58.44	29.83	17.14	14.16
2005	29.71	27.40	20.58	50.93	25.35	18.76	43.85
2006	29.92	26.67	24.69	48.60	21.12	31.60	38.36
2007	37.85	0.00	27.07	69.01	33.01	65.10	31.87
2008	38.62	38.96	27.98	35.81	42.65	47.78	46.69
2009	49.16	38.46	49.00	71.43	40.23	52.20	31.33
2010	50.25	49.38	43.09	81.81	65.63	34.32	41.62
2011	83.28	47.62	82.43	102.16	74.97	92.71	37.87
2012	77.85	90.91	62.78	102.59	115.84	94.10	21.81
2013	92.31	178.95	81.49	108.49	119.54	84.42	49.72
2014	43.31	36.04	48.60	43.66	38.40	38.69	22.75
2015	10.44	26.55	11.74	10.82	7.89	1.20	13.84
2016	10.57	25.86	14.45	9.11	6.32	4.88	8.42
2017	13.78	8.40	16.75	14.89	12.85	5.03	8.37
2018	11.84	0.00	12.81	16.37	13.52	7.52	5.20
2019	2.53	0.00	3.01	1.62	4.76	2.52	0.00
2020	0.00	0.00	0.00	0.00	0.00	0.00	0.00
