# Supplementary material for: The NIH public access policy did not harm biomedical journals
Source: PLoS Biol. 2019 Oct 23;17(10):e3000352. doi: 10.1371/journal.pbio.3000352 (PMC6808382; doi:10.1371/journal.pbio.3000352)
Supplement: S3 Data — OA, open access. (PDF) [file pbio.3000352.s007.pdf]

Supplementary Table 3. Birth rates for journals, expressed as births per 1000 journals, excluding open access

Year	All journals	AGRICUL	BIOMED	ENGTECH	NATSCI	PHYSCI	SOCSCI
1980	49.97	0.00	61.27	56.68	35.53	56.82	38.46
1981	52.13	0.00	58.89	57.47	56.37	28.78	50.26
1982	34.80	0.00	44.71	36.23	37.12	21.05	17.63
1983	41.94	46.51	56.43	34.97	40.27	30.93	24.75
1984	36.28	44.44	46.53	33.78	23.66	26.67	26.57
1985	32.49	21.28	39.77	29.41	18.99	22.80	30.59
1986	43.48	20.83	56.47	57.14	26.92	22.29	41.10
1987	39.33	61.22	45.77	36.14	32.32	28.04	37.28
1988	43.63	19.23	54.64	40.70	39.22	48.48	29.60
1989	36.72	18.87	44.03	42.02	28.30	23.12	34.98
1990	39.45	37.74	40.79	32.26	35.19	45.20	43.74
1991	38.75	0.00	40.91	44.27	33.99	51.35	26.67
1992	31.75	36.36	26.02	50.00	31.14	33.51	27.88
1993	29.21	35.09	29.55	50.00	31.93	32.50	9.04
1994	33.42	67.80	38.13	45.35	24.43	29.13	23.30
1995	34.51	32.26	36.13	45.75	27.03	37.74	26.32
1996	29.90	15.62	34.29	43.84	27.95	15.91	23.97
1997	29.63	30.77	27.73	40.16	22.69	22.37	36.79
1998	25.78	44.78	24.66	32.88	26.67	15.35	19.42
1999	27.32	0.00	33.85	28.09	25.97	17.28	19.08
2000	27.76	0.00	33.96	27.57	18.34	14.86	26.65
2001	23.51	0.00	22.15	32.43	13.91	21.01	24.50
2002	27.18	30.77	23.96	33.39	23.48	20.66	34.59
2003	21.37	0.00	21.45	27.44	16.19	22.27	14.64
2004	24.25	14.93	15.00	58.92	26.67	17.82	14.51
2005	27.47	29.41	19.53	46.40	22.22	17.54	40.58
2006	26.64	28.57	20.24	44.48	20.54	25.15	36.62
2007	30.89	0.00	22.89	45.66	30.34	51.14	31.46
2008	29.01	0.00	23.53	25.50	18.43	34.42	46.79
2009	38.47	42.86	30.52	70.03	29.30	45.61	29.72
2010	43.98	41.10	35.10	83.11	57.35	35.41	32.83
2011	75.65	53.33	65.19	106.62	76.66	88.67	34.48
2012	68.41	101.27	58.85	89.09	107.18	57.93	21.58
2013	88.02	197.67	76.64	105.75	117.82	75.91	48.41
2014	39.71	29.41	45.35	42.99	31.50	35.62	19.27
2015	7.31	29.13	7.96	7.28	4.36	1.37	8.95
2016	6.96	9.43	9.08	8.16	3.49	4.12	5.55
2017	12.26	0.00	14.69	15.32	9.70	5.52	8.84
2018	12.78	0.00	14.13	17.76	14.86	6.87	5.49
2019	2.76	0.00	3.31	1.75	5.21	2.74	0.00
2020	0.00	0.00	0.00	0.00	0.00	0.00	0.00
