# Supplementary material for: The NIH public access policy did not harm biomedical journals
Source: PLoS Biol. 2019 Oct 23;17(10):e3000352. doi: 10.1371/journal.pbio.3000352 (PMC6808382; doi:10.1371/journal.pbio.3000352)
Supplement: S4 Data — OA, open access. (PDF) [file pbio.3000352.s008.pdf]

Supplementary Table 4. Death rates for journals, expressed as deaths per 1000 journals, excluding open access

Year	All journals	AGRICUL	BIOMED	ENGTECH	NATSCI	PHYSCI	SOCSCI
1980	0.53	0.00	0.00	0.00	0.00	3.79	0.00
1981	0.50	0.00	0.00	0.00	0.00	3.60	0.00
1982	0.48	0.00	1.09	0.00	0.00	0.00	0.00
1983	0.46	0.00	1.04	0.00	0.00	0.00	0.00
1984	1.33	0.00	0.99	0.00	4.30	3.33	0.00
1985	0.00	0.00	0.00	0.00	0.00	0.00	0.00
1986	1.24	0.00	1.82	3.17	2.07	0.00	0.00
1987	1.19	0.00	2.59	0.00	2.02	0.00	0.00
1988	1.53	0.00	1.66	2.91	0.00	0.00	2.11
1989	3.31	18.87	3.14	0.00	9.43	0.00	0.00
1990	3.20	0.00	6.80	0.00	0.00	0.00	0.00
1991	1.71	0.00	2.19	2.60	0.00	2.70	1.90
1992	1.65	0.00	2.81	0.00	1.73	2.58	0.00
1993	1.28	0.00	2.06	0.00	0.00	2.50	0.00
1994	1.87	16.95	1.34	4.54	0.00	0.00	1.79
1995	1.82	0.00	1.29	2.18	3.18	0.00	1.75
1996	0.88	0.00	0.00	4.18	1.55	0.00	0.00
1997	1.71	0.00	1.21	2.01	1.51	2.24	3.34
1998	1.11	14.93	1.17	0.00	0.00	0.00	1.62
1999	4.33	14.49	3.44	9.36	2.89	0.00	4.77
2000	4.49	29.41	3.34	7.35	4.23	4.25	3.13
2001	6.20	15.15	7.56	7.21	6.95	4.20	6.13
2002	5.08	0.00	6.39	8.79	0.00	0.00	7.52
2003	6.96	0.00	9.94	8.58	4.05	0.00	5.86
2004	7.59	0.00	8.28	6.73	6.67	1.98	13.06
2005	5.30	0.00	4.11	3.20	3.92	9.75	11.59
2006	4.01	0.00	3.04	3.07	5.13	3.87	7.04
2007	5.99	0.00	7.96	5.89	1.26	5.68	8.21
2008	11.70	27.78	11.76	14.16	12.29	1.81	12.03
2009	7.96	0.00	9.21	8.40	7.33	5.26	6.46
2010	8.80	13.70	9.01	6.60	13.14	8.43	7.58
2011	6.22	0.00	4.62	6.13	9.15	11.49	7.39
2012	7.17	12.66	6.54	4.45	6.43	13.72	6.00
2013	7.30	11.63	7.87	7.19	4.87	10.22	7.08
2014	22.81	19.61	23.26	15.89	27.12	34.25	5.67
2015	4.99	0.00	5.31	3.64	6.97	5.47	1.12
2016	5.97	0.00	5.30	1.81	13.10	8.24	1.11
2017	1.32	0.00	1.51	0.90	0.88	1.38	2.21
2018	3.93	0.00	2.23	1.78	7.87	5.49	4.39
2019	0.16	0.00	0.37	0.00	0.00	0.00	0.00
2020	0.00	0.00	0.00	0.00	0.00	0.00	0.00
