# Supplementary material for: The NIH public access policy did not harm biomedical journals
Source: PLoS Biol. 2019 Oct 23;17(10):e3000352. doi: 10.1371/journal.pbio.3000352 (PMC6808382; doi:10.1371/journal.pbio.3000352)
Supplement: S5 Data — (PDF) [file pbio.3000352.s009.pdf]

	year	total	birth	death	totallag	birthrate	deathrate	birthnet
1	1980	2022	97	1	1926	50.3634476	0.5192108	96
2	1981	2125	104	1	2022	51.4342235	0.49455984	103
3	1982	2200	76	1	2125	35.7647059	0.47058824	75
4	1983	2291	92	1	2200	41.8181818	0.45454545	91
5	1984	2372	84	3	2291	36.6652117	1.30947185	81
6	1985	2448	76	0	2372	32.0404722	0	76
7	1986	2552	107	3	2448	43.7091503	1.2254902	104
8	1987	2649	100	3	2552	39.184953	1.17554859	97
9	1988	2761	116	4	2649	43.7901095	1.51000378	112
10	1989	2854	102	9	2761	36.9431365	3.25968852	93
11	1990	2958	113	9	2854	39.5935529	3.15346882	104
12	1991	3068	115	5	2958	38.87762	1.6903313	110
13	1992	3164	101	5	3068	32.9204694	1.62972621	96
14	1993	3257	97	4	3164	30.6573957	1.2642225	93
15	1994	3362	111	6	3257	34.0804421	1.84218606	105
16	1995	3477	121	6	3362	35.9904819	1.78465199	115
17	1996	3581	107	3	3477	30.7736555	0.86281277	104
18	1997	3685	110	6	3581	30.7176766	1.67550963	104
19	1998	3776	95	4	3685	25.78019	1.08548168	91
20	1999	3866	106	16	3776	28.0720339	4.23728814	90
21	2000	3959	110	17	3866	28.4531816	4.39730988	93
22	2001	4032	97	24	3959	24.5011367	6.0621369	73
23	2002	4124	112	20	4032	27.7777778	4.96031746	92
24	2003	4195	100	29	4124	24.2483026	7.03200776	71
25	2004	4268	105	32	4195	25.0297974	7.62812872	73
26	2005	4372	127	23	4268	29.7563261	5.38894096	104
27	2006	4486	130	16	4372	29.7346752	3.65965233	114
28	2007	4629	170	27	4486	37.8956754	6.01872492	143
29	2008	4753	178	54	4629	38.4532296	11.6655865	124
30	2009	4947	233	39	4753	49.0216705	8.20534399	194
31	2010	5155	249	41	4947	50.3335355	8.28785122	208
32	2011	5553	429	31	5155	83.2201746	6.01357905	398
33	2012	5948	432	37	5553	77.7957861	6.66306501	395
34	2013	6452	547	43	5948	91.9636853	7.22932078	504
35	2014	6596	280	136	6452	43.3973962	21.0787353	144
36	2015	6608	69	57	6596	10.4608854	8.64160097	12
37	2016	6594	70	84	6608	10.5932203	12.7118644	-14
38	2017	6665	91	20	6594	13.8004246	3.03306036	71
39	2018	6700	79	44	6665	11.8529632	6.60165041	35
40	2019	6716	17	1	6700	2.53731343	0.14925373	16
41	2020	6716	0	0	6716	0	0	0
