# Supplementary figures and images for: The NIH public access policy did not harm biomedical journals
Source: PLoS Biol. 2019 Oct 23;17(10):e3000352. doi: 10.1371/journal.pbio.3000352 (PMC6808382; doi:10.1371/journal.pbio.3000352)

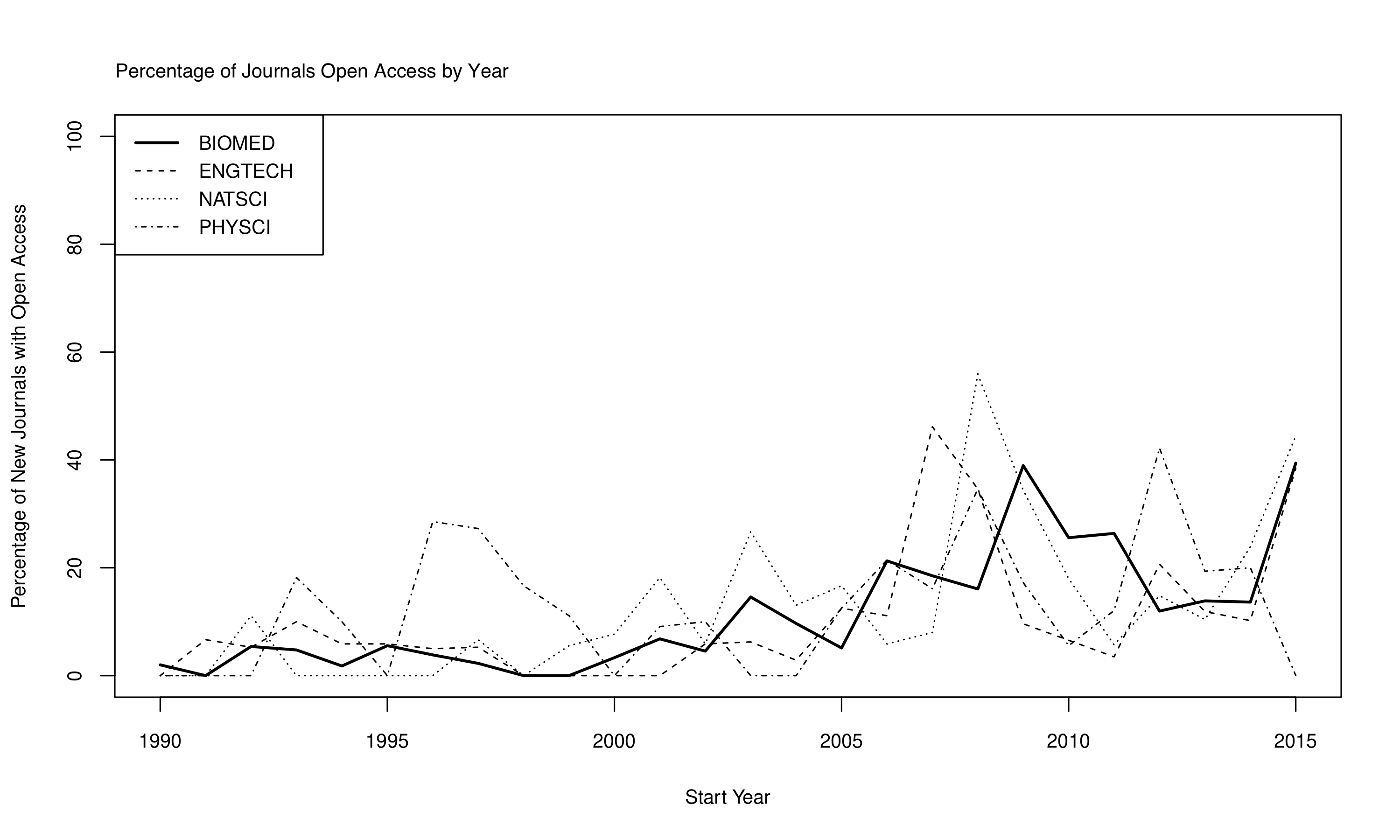

Supplement: S1 Fig — OA, open access. (TIF) [file pbio.3000352.s010.tif]

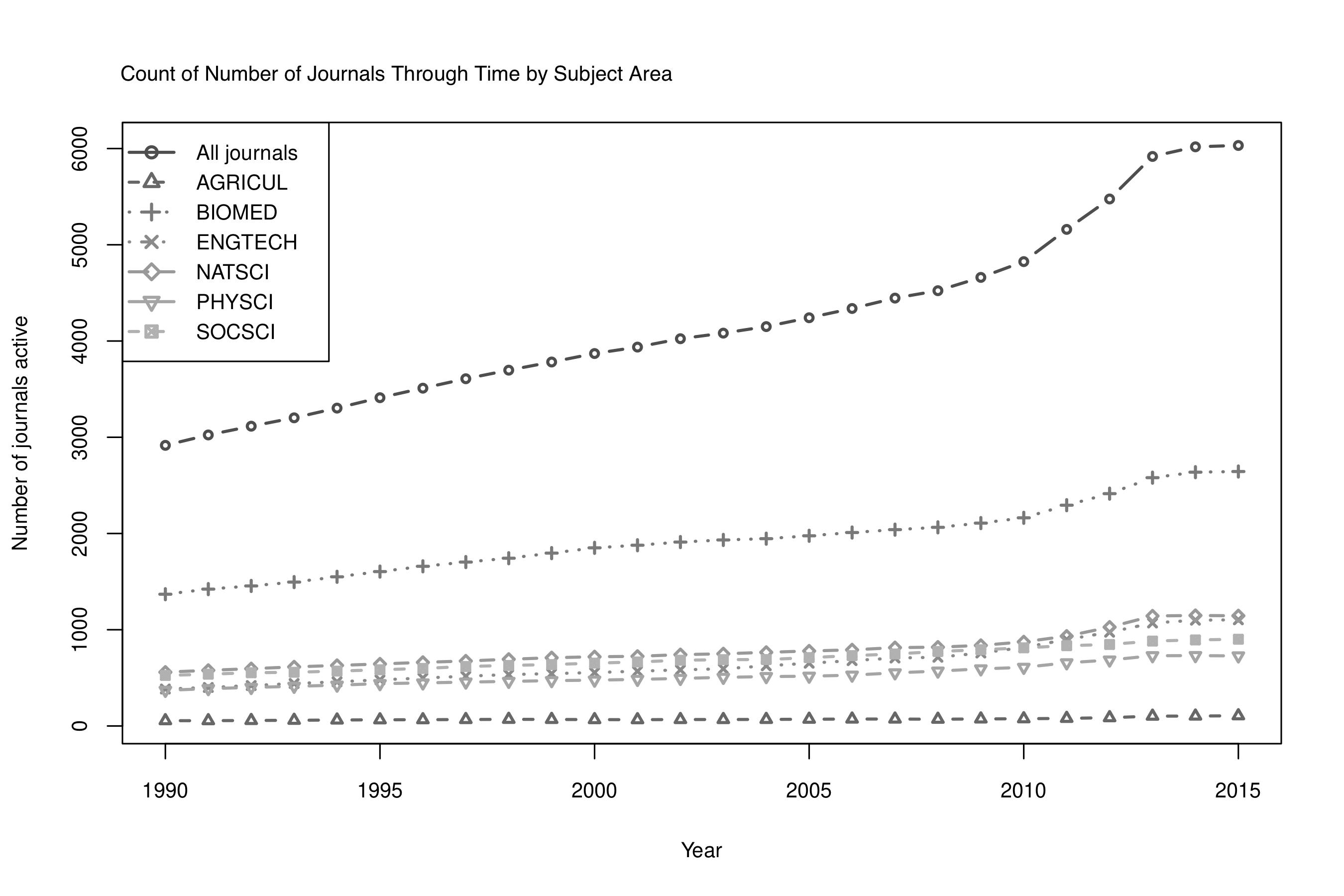

Supplement: S2 Fig — OA, open access. (TIF) [file pbio.3000352.s011.tif]

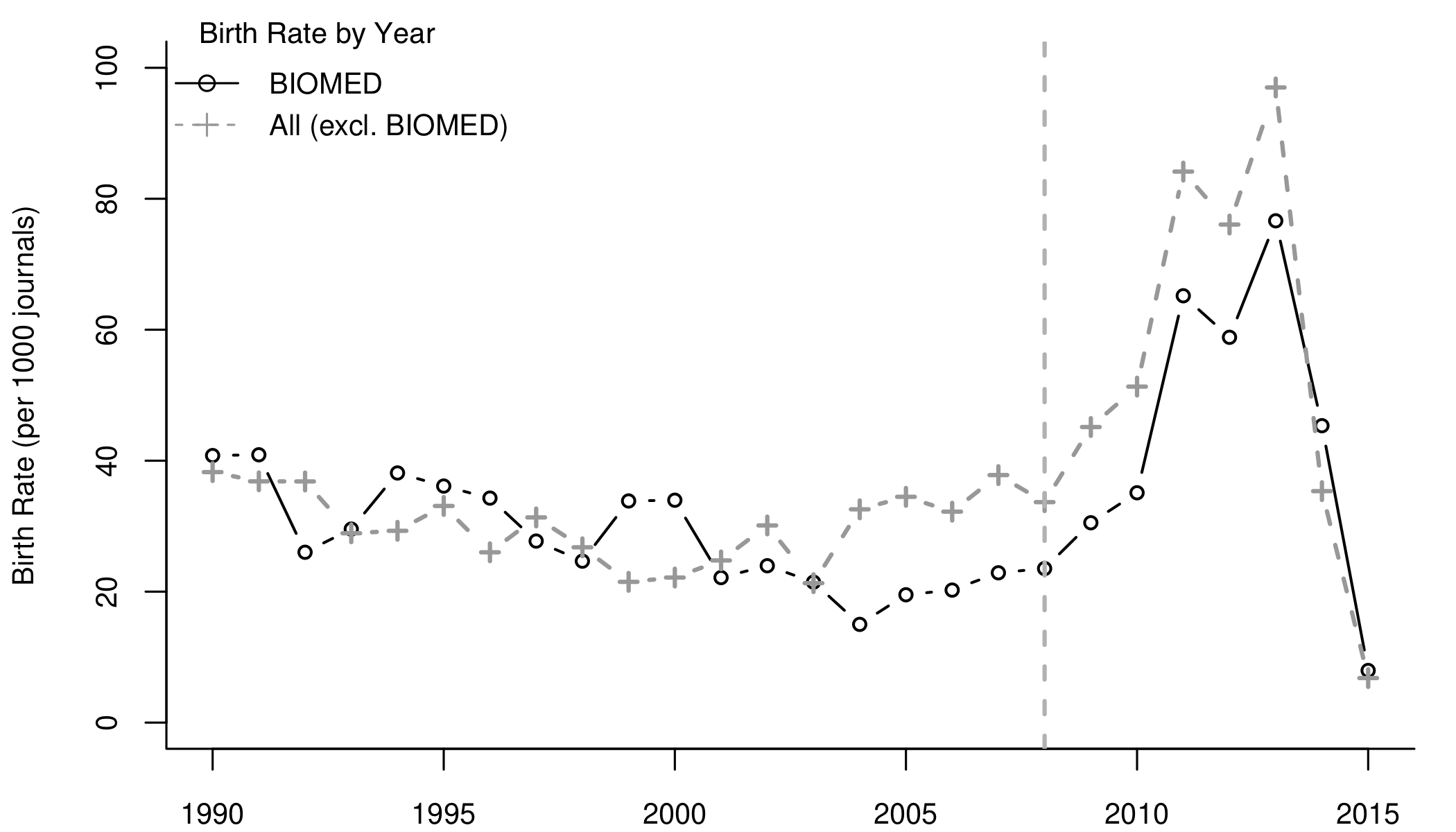

Supplement: S3 Fig — OA, open access. (TIF) [file pbio.3000352.s012.tif]

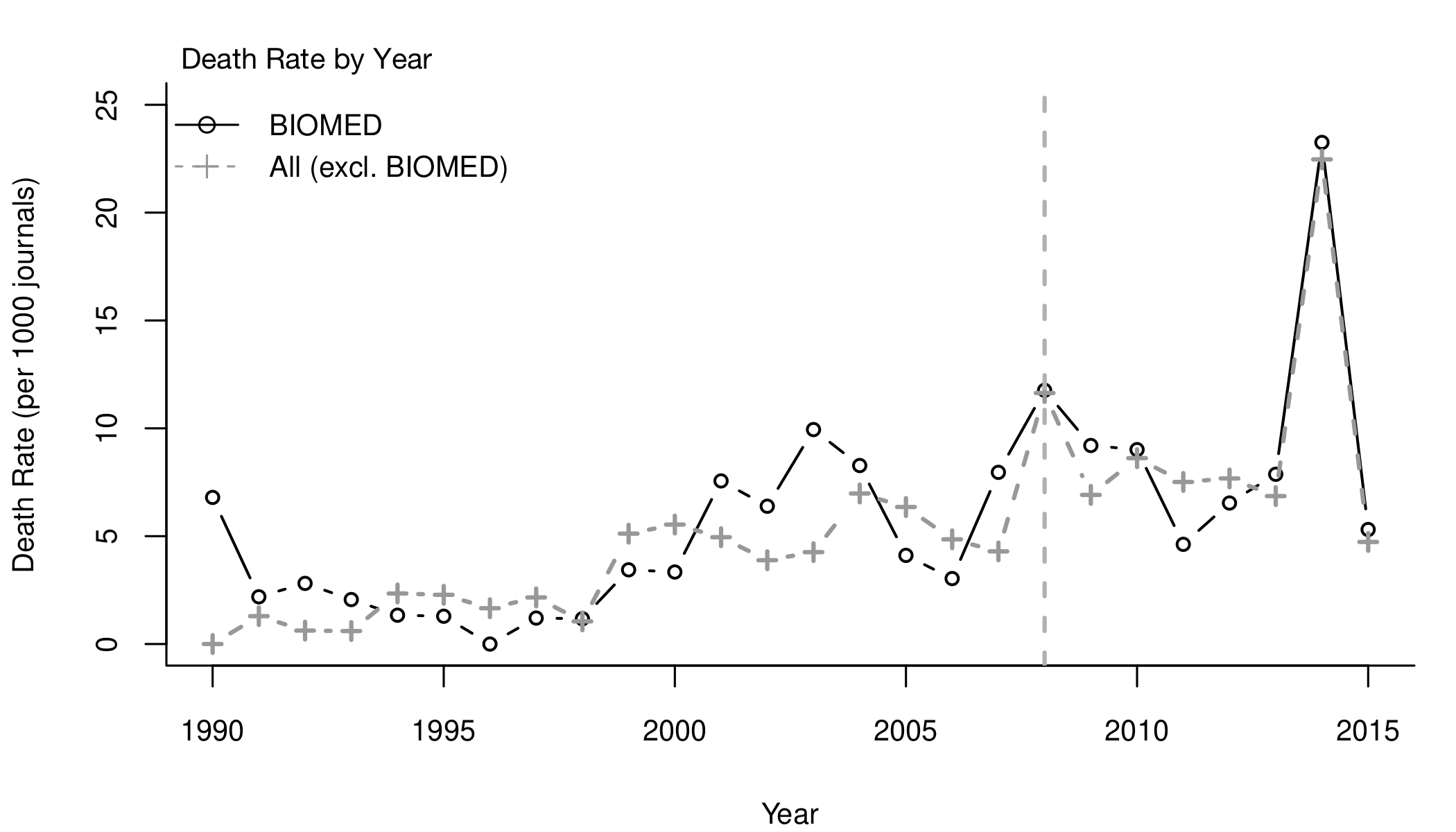

Supplement: S4 Fig — OA, open access. (TIF) [file pbio.3000352.s013.tif]

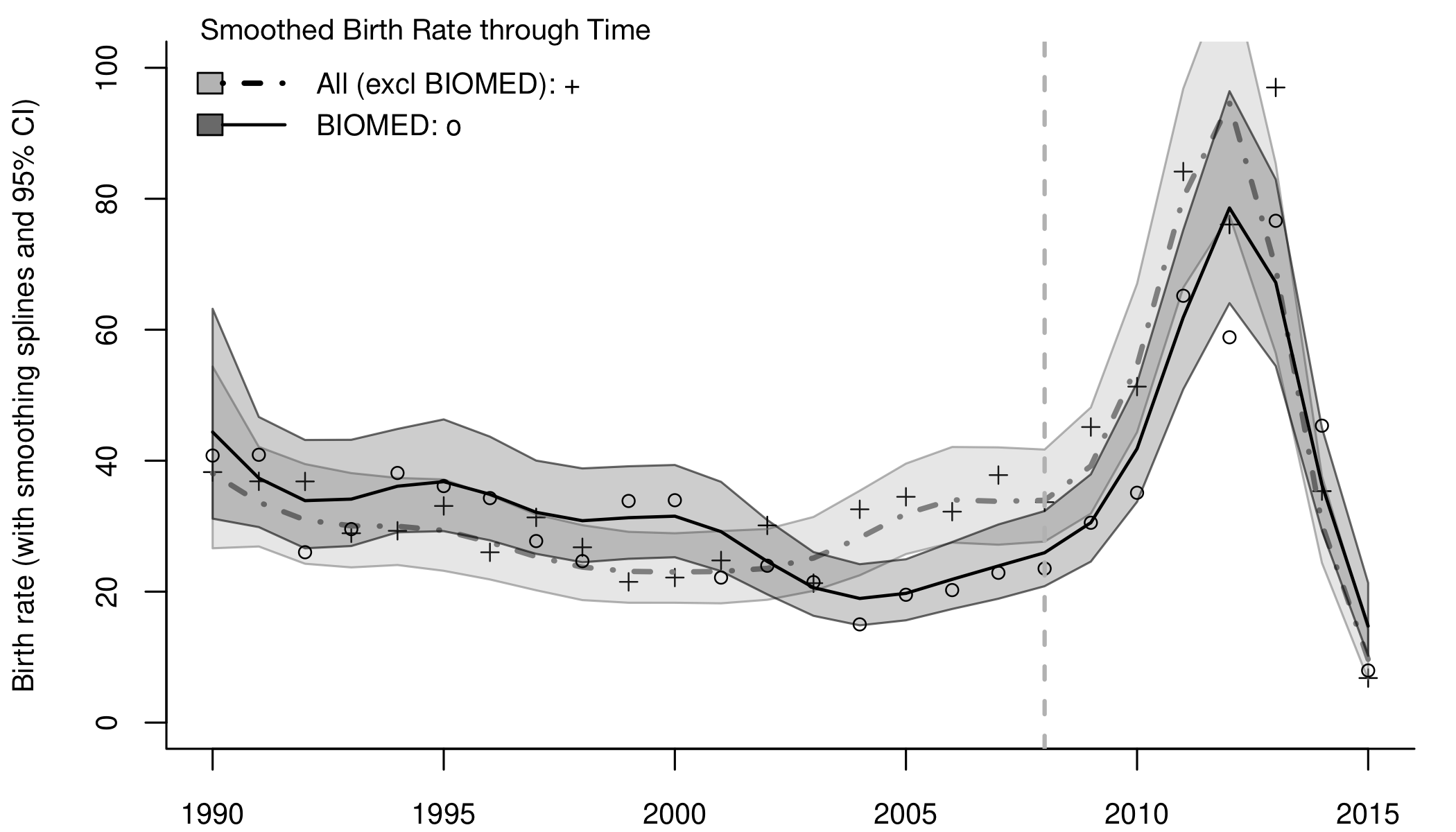

Supplement: S5 Fig — OA, open access. (TIF) [file pbio.3000352.s014.tif]

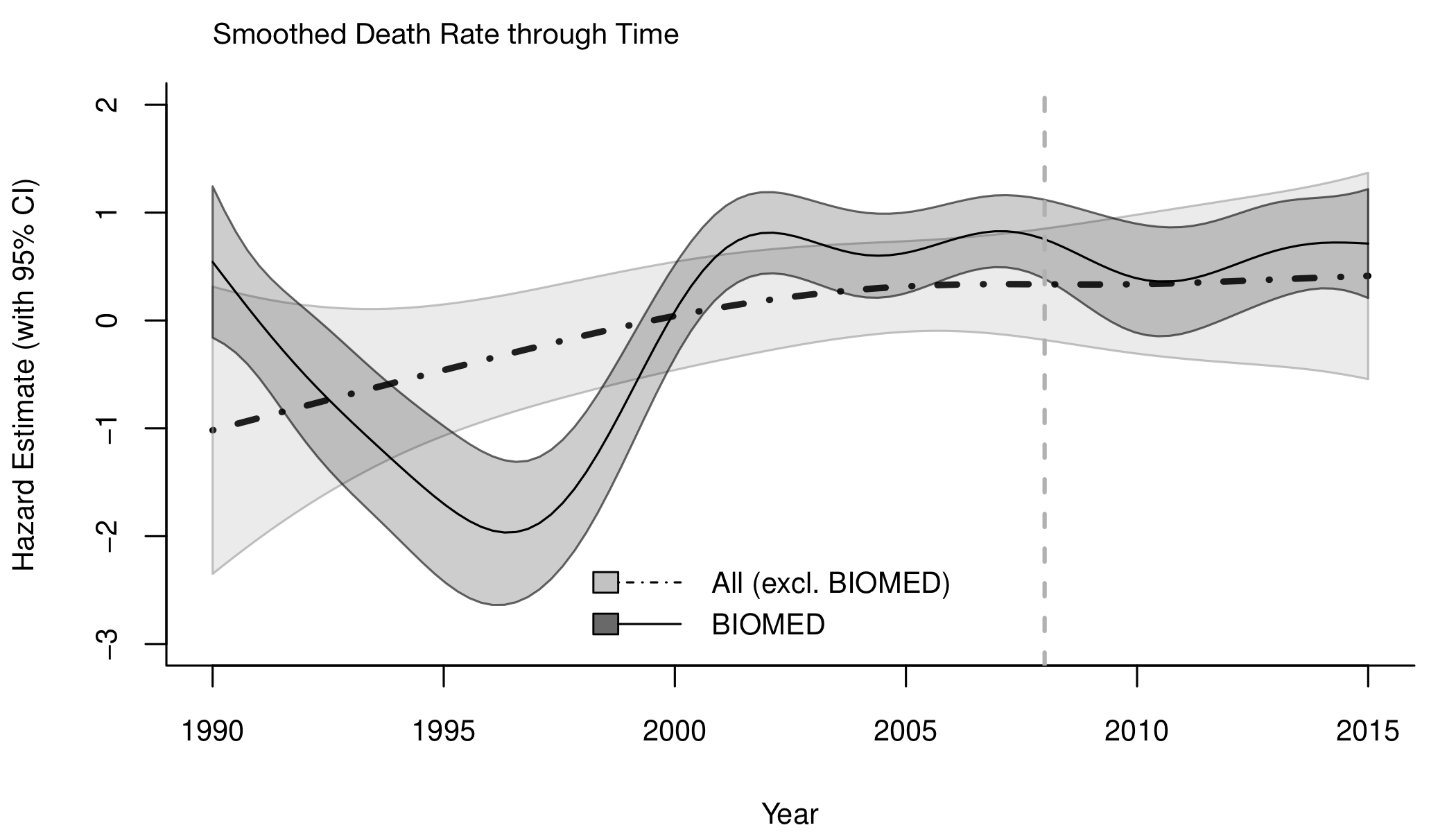

Supplement: S6 Fig — OA, open access. (TIF) [file pbio.3000352.s015.tif]

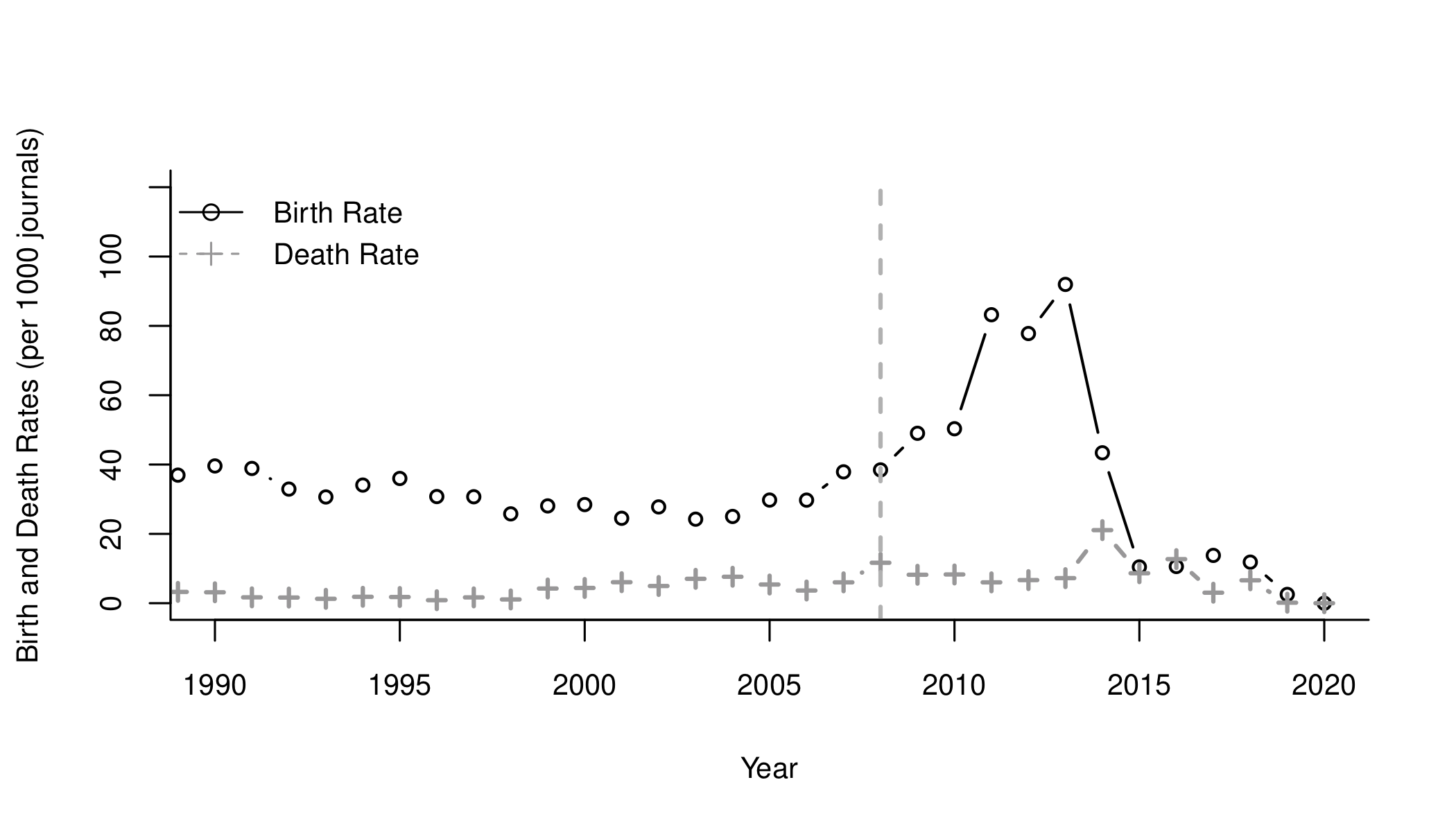

Supplement: S7 Fig — (TIFF) [file pbio.3000352.s016.tiff]
